# Recent advances in palladium-catalysed asymmetric 1,4–additions of arylboronic acids to conjugated enones and chromones

**DOI:** 10.3762/bjoc.17.84

**Published:** 2021-05-10

**Authors:** Jan Bartáček, Jan Svoboda, Martin Kocúrik, Jaroslav Pochobradský, Alexander Čegan, Miloš Sedlák, Jiří Váňa

**Affiliations:** 1 Institute of Organic Chemistry and Technology, Faculty of Chemical Technology, University of Pardubice, Studentská 573, 532 10 Pardubice, Czech Republic,; 2 Department of Biological and Biochemical Sciences, Faculty of Chemical Technology, University of Pardubice, Studentská 573, 532 10 Pardubice, Czech Republic

**Keywords:** asymmetric reaction, boronic acid, conjugated enones, chromones, enantioselective catalysis, Michael addition, Pd complexes

## Abstract

The transition metal (palladium)-catalysed asymmetric 1,4-addition of arylboronic acids to conjugated enones belong to the most important and emerging strategies for the construction of C–C bonds in an asymmetric fashion. This review covers known catalytic systems used for this transformation. For clarity, we are using the type of ligand as a sorting criterion. Finally, we attempted to create a flowchart facilitating the selection of a suitable ligand for a given combination of enone and arylboronic acid.

## Introduction

The asymmetric 1,4-addition of arylboronic acids to conjugated cyclic enones and chromones is a very important reaction nowadays. For illustration, the addition products are very promising in medicinal chemistry research [1–7] and in natural products total syntheses [8–16]. Chiral complexes of Rh [17–24] and Pd usually catalyse the reaction, however, palladium holds a special place in this area. There are several review articles partially covering this topic [25–31]. However, a comprehensive review is missing. In the following sections, we attempt to fill this gap. As a sorting criterion, the type of ligand (phosphines, NHC-carbenes, bisoxazolines, pyridine-oxazolines, and miscellaneous) is used.

## Review

### Catalytic systems based on phosphine ligands

A pioneering work on the enantioselective addition of boron-derived carbon nucleophiles to cyclic enones was published by the group of Miyaura et al. in 2005 [32]. Specifically, they have dealt with the addition of potassium aryltrifluoroborates to conjugated cyclic enones differing in ring size [32]. The catalysts **PdL1a**,**b** exhibited great conversion and enantioselectivities (up to 99% and up to 96% ee) for various combinations of nucleophiles and enones (Table 1). The authors also studied the possibility of the addition of boronic acids. The reaction of phenylboronic acid with 2-cyclohexenone catalysed by 5% of achiral [Pd(dppe)(PhCN)_2_](BF_4_)_2_ at −5 °C gave the product in 21% yield. When 1 equiv of BF_3_·OEt_2_ was added, the yield was increased to 74%. This result led to the conclusion that in this catalytic system, much better results were obtained when aryltrifluoroborates are used. The system also worked well for linear enone electrophiles (entries 12–20, Table 1). The main disadvantage of this approach is the necessity of sub-zero temperatures [32–33].

**Table 1:** First example of asymmetric addition of organoboron reagents to cyclic enones [32–33].

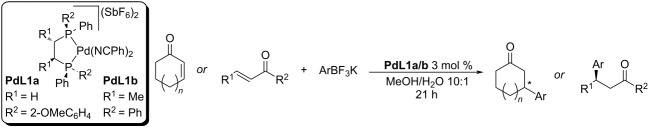							

entry	cyclic substrate		Ar	cat.	temp. (°C)	yield (%)	ee (%)
	*n*						

1	0		Ph	**PdL1a**	−5	60	95 (*S*)
2	1		Ph	**PdL1b**	−15	95	93 (*R*)
3	1		4-MeO-C_6_H_4_	**PdL1b**	−5	89	85 (*R*)
4	1		3-MeO-C_6_H_4_	**PdL1b**	−15	97	95 (*R*)
5	1		4-Me-C_6_H_4_	**PdL1b**	−5	70	90 (*R*)
6	1		3-Me-C_6_H_4_	**PdL1b**	−5	96	93 (*R*)
7	1		4-F-C_6_H_4_	**PdL1b**	−5	99	92 (*R*)
8	1		3-F-C_6_H_4_	**PdL1b**	−15	81	96 (*R*)
9	1		4-CF_3_-C_6_H_4_	**PdL1b**	−5	33	87 (*R*)
10	1		4-CF_3_-C_6_H_4_	**PdL1b**	−5	66^a^	92^a^ (*R*)
11	2		Ph	**PdL1b**	−15	91	89 (*R*)

	acyclic substrate						
	R^1^	R^2^					

12	*n*-C_5_H_11_	iPr	Ph	**PdL1a**	−15	93	87
13	*n*-C_5_H_11_	Cy	Ph	**PdL1a**	−15	98	88
14	*n*-C_5_H_11_	Ph	Ph	**PdL1a**	−15	99	89
15	iPr	Me	3-MeO-C_6_H_4_	**PdL1a**	−5	65	83
16	Cy	Me	Ph	**PdL1a**	−5	22	78
17	Ph	Me	3-MeO-C_6_H_4_	**PdL1a**	0	90	95
18	Ph	*n*-Bu	3-MeO-C_6_H_4_	**PdL1a**	5	91	99
19	Ph	Ph	3-MeO-C_6_H_4_	**PdL1a**	−5	94	97
20	2-naphthyl	Me	3-MeO-C_6_H_4_	**PdL1a**	0	73	96

^a^No water added.

A follow-up report of the Miyaura group in 2007 provided an experimental protocol that allowed the addition of arylboronic acids instead of aryltrifluoroborates [34]. The previously used catalysts **PdL1a**,**b** were combined with additional silver salts (AgBF_4_ or AgSbF_6_) that greatly accelerated the transmetalation of the boronic acid to Pd. This enhanced catalytic system showed a great turnover number (TON) up to 9,900. The authors described additions to cyclic substrates with high yields (90–99%) and enantioselectivities (89–94% ee; entries 1–5, Table 2). Also, a library of linear enones was tested giving excellent yields and enantioselectivities in most of the cases (with up to 99% yield and 99% ee; entries 6–24, Table 2). Several substrates did not even require the addition of Ag(I) salts to achieve high yields (entries 7, 10, 12, 17, 22, and 23, Table 2) [34–35].

**Table 2:** Addition of arylboronic acids to enones accelerated by silver salts [34–35].

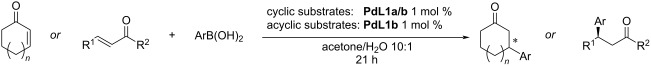							

entry	cyclic substrates		Ar	additive (equiv)	temp. (°C)	yield (%)	ee (%)
	*n* (catalyst)						

1	0 (**PdL1b**)		Ph	AgBF_4_	0	94	94 (S)
2	1 (**PdL1a**)		Ph	AgBF_4_	0	90	92 (*R*)
3	1 (**PdL1a**)		Ph	AgBF_4_ (0.05)	20	99^a^	89 (*R*)
4	1 (**PdL1a**)		3-MeO-C_6_H_4_	AgBF_4_ (0.05)	20	98^a^	91 (*R*)
5	2 (**PdL1a**)		Ph	AgBF_4_	0	92	89 (*R*)

	acyclic substrates						
	R^1^	R^2^					

6	Ph	Ph	4-Me-C_6_H_4_	AgBF_4_ (0.1)	20	73	95
7	Ph	Me	3-Cl-C_6_H_4_	**–**	25	90	93
8	Ph	Me	3-MeO-C_6_H_4_	AgBF_4_ (0.1)	0	96	95
9	Ph	Me	4-MeO-C_6_H_4_	AgBF_4_ (0.1)	0	75	94
10	Ph	Me	3,4-(CH_2_O_2_)-C_6_H_3_	**–**	0	77	95
11	Ph	Me	4-MeS-C_6_H_4_	AgBF_4_ (0.1)	25	<10	–
12	Ph	Me	4-Ac-C_6_H_4_	**–**	0	95	93
13	Ph	*n*-Bu	3-MeO-C_6_H_4_	AgBF_4_ (0.1)	0	66	99
14	Ph	iPr	3-MeO-C_6_H_4_	AgBF_4_ (0.1)	0	80	95
15	Ph	Cy	3-MeO-C_6_H_4_	AgSbF_6_ (0.05)	0	93	95
16	Ph	Ph	3-MeO-C_6_H_4_	AgBF_4_ (0.1)	0	86	97
17	Ph	Ph	4-Me-C_6_H_4_	**–**	0	91	95
18	Ph	4-MeO-C_6_H_4_	3-MeO-C_6_H_4_	AgSbF_6_ (0.1)	0	73	95
19	Ph	3-NO_2_-C_6_H_4_	3-MeO-C_6_H_4_	AgSbF_6_ (0.2)	0	44	92
20	4-MeO-C_6_H_4_	Ph	3-MeO-C_6_H_4_	AgBF_4_ (0.1)	0	75	99
21	2-naphthyl	Me	3-MeO-C_6_H_4_	AgBF_4_ (0.1)	0	99	96
22	2-BnO-5-Me-C_6_H_3_	Me	Ph	–	0	97	96
23		Ph	Ph	–	0	86	98
24	*n*-C_5_H_11_	Me	Ph	AgBF_4_	0	99	80

^a^Reaction time: 48 h.

An interesting finding was that β-(2-hydroxyaryl)enones underwent cyclization to ketals (chromanols) after the addition of boronic acid. The prepared chromanols afforded the chromenes through elimination upon treatment with *p*-TsOH. A series of different β-(2-hydroxyaryl)enones and boronic acids was tested and provided the substituted chromenes in excellent yields (89–94%) and enantioselectivities (95–99% ee; Table 3). It is worth mentioning that a free phenolic hydroxy group did not interfere with the Pd complex and did not affect the enantioselectivity of the reaction.

**Table 3:** Synthesis of chromenes by the 1,4-addition of boronic acids to β-(2-hydroxyaryl)enones [34].

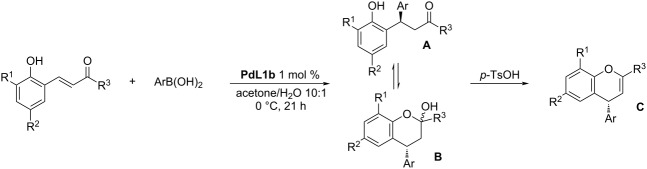								

entry	R^1^	R^2^	R^3^	Ar	additive (equiv)	yield **A** + **B** (%) ratio **A**/**B**	yield **C** (%)	ee **C** (%)

1	H	Me	Me	Ph	–	99 (1:13)	90	96
2	H	Me	Me	4-MeO-C_6_H_4_	AgBF_4_ (0.1)	96 (1:13)	90	97
3	H	Me	Me	3-MeO-C_6_H_4_	AgBF_4_ (0.1)	96 (1:13)	94	97
4	H	Me	Me	3,4-(CH_2_O_2_)-C_6_H_3_	AgBF_4_ (0.1)	99 (1:16)	89	98
5	H	Me	Me	4-Me-C_6_H_4_	–	99 (1:13)	94	97
6	H	Me	Me	4-Ac-C_6_H_4_	AgBF_4_ (0.1)	99 (1:16)	90	96
7	H	H	Ph	Ph	–	99 (2:1)	92	99
8	H	OMe	Me	Ph	–	99 (1:16)	94	95
9	*t*-Bu	*t*-Bu	Me	Ph	–	94 (1:99)	90	–

**Scheme 1:**
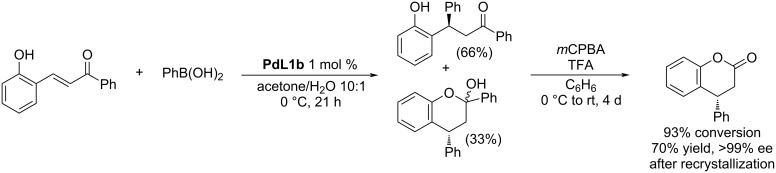
Synthesis of optically pure 4-phenylchroman-2-one [34].

The authors also demonstrated that the product mixture obtained after the addition of the boronic acid to the β-(2-hydroxyaryl)enone could be oxidized to afford optically pure 4-phenylchroman-2-one (Scheme 1).

Also in 2007, Miyaura and co-workers presented the synthesis of enantioenriched 1-aryl-1*H*-indenes by a tandem 1,4-addition of arylboronic acids to enones and aldol condensation [36]. The catalytic system for this transformation was adapted from earlier works [34,36] and included the addition of a 42% aqueous solution of HBF_4_ that facilitated consequent cyclization. A series of various β-(2-acylphenyl)enones and arylboronic acids was tested. Almost every combination provided the product in an excellent yield (60–99%) and enantioselectivity (up to 97% ee; Table 4), the only exception being the addition of an *ortho*-substituted boronic acid (entry 5, Table 4) [36].

**Table 4:** Synthesis of enantiomerically enriched 1-aryl-1*H*-indenes [36].

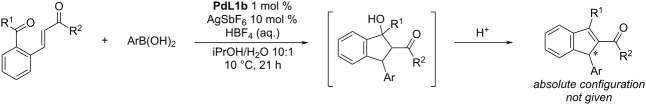					

entry	R^1^	R^2^	Ar	yield (%)	ee (%)

1	Me	Me	Ph	95	90
2	Me	Me	4-Cl-C_6_H_4_	91	90
3	Me	Me	3-Cl-C_6_H_4_	88	91
4	Me	Me	4-Me-C_6_H_4_	94	93
5	Me	Me	2-MeO-C_6_H_4_	60	24
6	Me	Me	3-MeO-C_6_H_4_	91	93
7	Me	Me	4-MeO-C_6_H_4_	90	96
8	Me	Me	3,4-(CH_2_O_2_)-C_6_H_3_	76	93
9	Me	Me	4-(4-MeO-C_6_H_4_)-C_6_H_4_	91	97
10	Me	Me	3-BnO-C_6_H_4_	90	94
11	Ph	Me	4-MeO-C_6_H_4_	99	92
12	Ph	4-MeO-C_6_H_4_	4-MeO-C_6_H_4_	79	90
13	Ph	4-MeO-C_6_H_4_	3,4-(CH_2_O_2_)-C_6_H_3_	81	90
14	Me	Et	4-MeO-C_6_H_4_	99	93
15	H	Me	4-MeO-C_6_H_4_	60	90

In 2008, the same group further expanded the substrate scope of the addition reaction to electron-rich chalcones. The products obtained after the addition reaction with arylboronic acids were further subjected to a regioselective Bayer–Villiger oxidation (Table 5) [3].

**Table 5:** Stepwise addition of arylboronic acids to electron-rich chalcones and Bayer–Villiger oxidation [3].

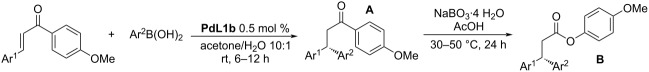						

entry	Ar^1^	Ar^2^	yield **A** (%)	ee **A** (%)	yield **B** (%)	ee **B** (%)

1	Ph	3-MeO-C_6_H_4_	99	95	73	95
2	4-iPr-C_6_H_4_	3-MeO-C_6_H_4_	90	95	0	–
3	4-MeO-C_6_H_4_	3,4-diMeO-C_6_H_4_	86	95	72	97
4	3,4-(CH_2_O_2_)-C_6_H_3_	3,4-diMeO-C_6_H_4_	74^a^	97	67	95
5	2-BnO-5-Me-C_6_H_3_	Ph	91 (83)^b^	95 (99)^b^	–	

^a^Reaction performed in MeOH/water 10:1 instead of acetone/water 10:1; ^b^after recrystallization.

An enhanced protocol for the synthesis of 4-aryldihydrocoumarins (Table 6) was also presented [3], which was already mentioned above (Scheme 1) [34].

**Table 6:** Synthesis of 4-aryldihydrocoumarins by stepwise 1,4-addition and Bayer–Villiger oxidation [3].

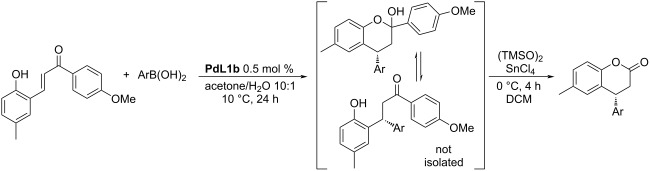			

entry	Ar	yield (%)	ee (%)

1	Ph	83	96
2	4-MeO-C_6_H_4_	75	98
3	3,4-(CH_2_O_2_)-C_6_H_3_	70	97
4	4-MeO-3,5-diMe-C_6_H_2_	74	97

**Scheme 2:**
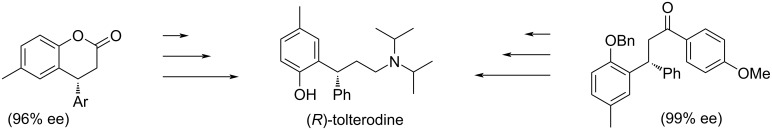
Synthesis of (*R*)-tolterodine [3].

Both presented methods were used in the synthesis of an antimuscarinic drug (*R*)-tolterodine (Scheme 2) [3].

**Scheme 3:**
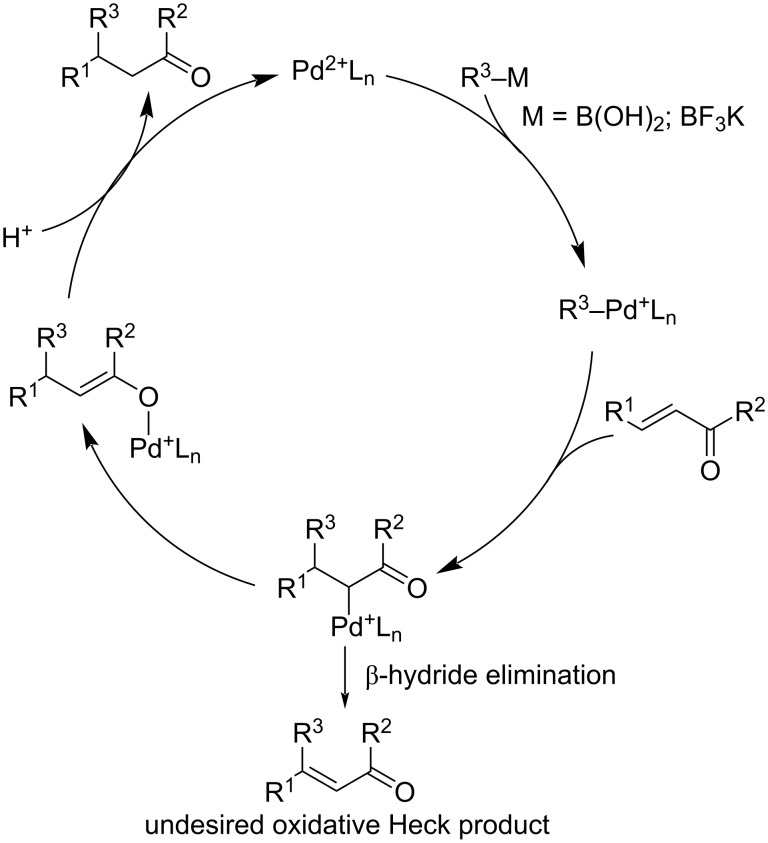
Catalytic cycle of the Pd(II)-catalysed 1,4-addition of organoboron reagents to enones [3,26,35].

A plausible catalytic cycle has been proposed (Scheme 3). The usual cross-coupling of an organoboron to Pd(0) requires a base. In the case of Pd(II) this reaction smoothly progresses under neutral conditions. The authors postulated that the vacancy on the square-planar Pd(II) species allows a faster alkene insertion in comparison to Pd(0). The cationic Pd(II) enolate exists as a dynamic mixture of *C*- and *O*-bound enolate and is highly susceptible to hydrolysis. This means that in the presence of water, it is selectively converted to the 1,4-addition product instead of undergoing a β-hydride elimination leading to an oxidative Heck product [3,26,35].

In 2005, one month after the very first report of the addition of aryltrifluoroborates to enones by Miyaura [32], the Minnaard group reported a protocol for the addition of boronic acids to enones [37]. At first, they tested the combination of Pd(OAc)_2_ with triflic acid (TfOH) to obtain a Pd(II) complex with a weakly coordinating anion that is necessary for a fast Pd–C bond cleavage and thus avoiding the undesired β-hydride elimination. However, the obtained yields were inconsistent. The usage of Pd(TFA)_2_ led to a better reproducibility of the results. From the various diphosphine ligands tested, (*R*,*R*)-MeDuPhos (**L2**) was identified as the one leading to the best level of enantioselectivity (up to 99% yield and up to 99% ee; Table 7) [37].

**Table 7:** First report of the Pd-catalysed enantioselective addition of boronic acids to cyclic enones [37].

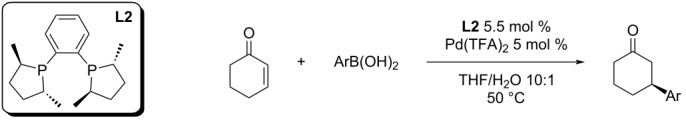				

entry	Ar	time (h)	yield (%)	ee (%)

1	Ph	6	80	98
2	2-MeO-C_6_H_4_	18	80	99
3	2-Me-C_6_H_4_	18	>99	99
4	3-Me-C_6_H_4_	18	>99	97
5	3-MeO-C_6_H_4_	18	98	97
6	4-Me-C_6_H_4_	18	90	98
7	3-NO_2_-C_6_H_4_	24	0	–
8	3-Cl-C_6_H_4_	24	40	98

Furthermore, water was discovered to be a crucial additive in the reaction, increasing the yield without impact on the enantioselectivity [37]. The presented catalytic system worked well in the case of electron-rich arylboronic acids (entries 1–6, Table 7). Electron-poor arylboronic acids reacted much slower or did not react at all due to the slow transmetalation to Pd (entries 7 and 8, Table 7) [37]. The addition of phenylboronic acid (or aprotic triphenylboroxine with slow addition of water to the reaction mixture) was also tested in combination with enones differing in ring size, unsaturated lactone, *N*-protected dihydropyridone and one example of a linear substrate. In all cases a decreased reactivity was observed, however, good to excellent enantioselectivity levels were maintained (81–99% ee; Table 8) [37].

**Table 8:** Addition of boron-derived *C*-nucleophiles to cyclic enones, catalysed by **L2**/Pd(TFA)_2_ [37].

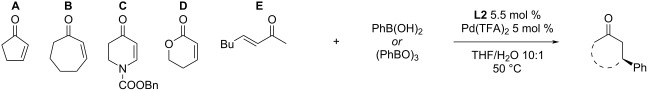					

entry	substrate	*C*-nucleophile	time (h)	yield (%)	ee (%)

1	**A**	PhB(OH)_2_	6	75	82
2	**B**	PhB(OH)_2_	18	55	86
3	**C**	PhB(OH)_2_	22	60	>99
4	**D**	(PhBO)_3_ (slow addition of water)	5	75	94
5	**E**	(PhBO)_3_ (slow addition of water)	18	45(60%^a^)	81

^a^Conversion.

**Scheme 4:**
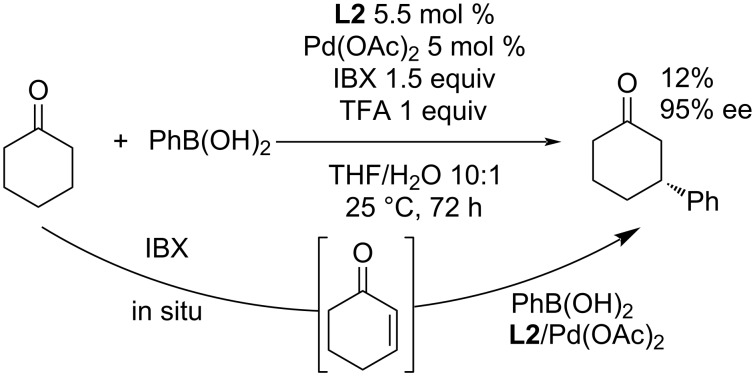
Enantioselective β-arylation of cyclohexanone [38].

To our best knowledge, at this time only one method for the enantioselective β-arylation of cyclic ketones is known [38]. In 2017, Hu et al. presented the possibility of an enantioselective β-arylation of cyclohexanone using the above mentioned ligand **L2**. Cyclohexanone was in situ oxidized by 2-iodoxybenzoic acid (IBX) to 2-cyclohexenone, that subsequently underwent addition of phenylboronic acid (Scheme 4). The complex **L2**/Pd(OAc)_2_ was used to obtain the product with excellent enantioselectivity (95% ee) but only poor yield (12%) (Scheme 4) [38].

**Scheme 5:**
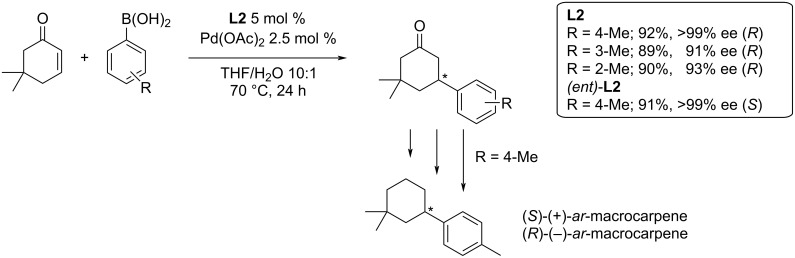
Application of **L2**/Pd(OAc)_2_ in the total synthesis of terpenes [8].

A catalytic system based on **L2**/Pd(OAc)_2_ was recently used by Khatua et al. for the synthesis of *ar*-macrocarpenes with excellent yields and enantioselectivities (89–92%; 91–99% ee; Scheme 5) [8].

In 2007, the group of Ito described the application of ferrocenylphosphines for the palladium-catalysed addition of arylboronic acids to 2-cyclohexenone at various temperatures giving the products with high conversions but only very low enantioselectivities (25–71% ee; Table 9) [39].

**Table 9:** Asymmetric addition of arylboronic acids to 2-cyclohexenone catalysed by **L3**/Pd(dba)_2_ [39].

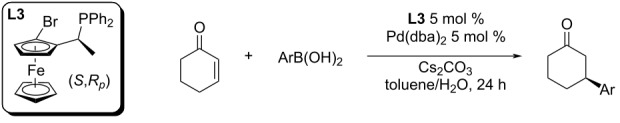				

entry	Ar	temp. (°C)	yield (%)	ee (%)

1	Ph	80	82	42
2	Ph	60	83	46
3	Ph	25	79	66
4	4-Me-C_6_H_4_	80	88	61
5	4-Me-C_6_H_4_	25	90	71
6	2-Me-C_6_H_4_	80	93	25
7	3-Me-C_6_H_4_	80	63	58

**Scheme 6:**
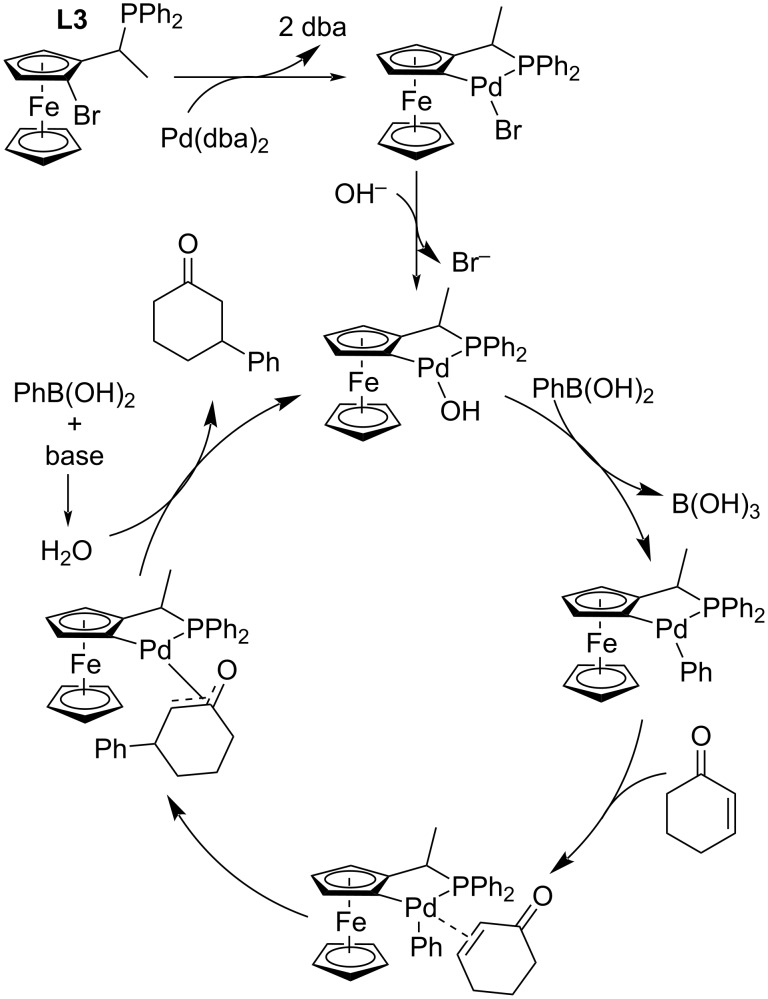
Plausible catalytic cycle for the addition of phenylboronic acid to 2-cyclohexenone catalysed by **L3**/Pd(dba)_2_ [40].

The same group continued their work on this catalytic system under different reaction conditions with the cheaper base K_2_CO_3_ and without the addition of water. The observed yields were excellent (45–94%) although the enantioselectivities were only average to poor (4–79% ee; entries 1–9, Table 10). Also several linear enones were tested giving the products with varying yields (53–99%) and only moderate enantioselectivities (42–52% ee; entries 10–13, Table 10) [40]. Additionally, the authors proposed a plausible catalytic cycle for the reaction (Scheme 6) [40].

**Table 10:** Additions to different enones catalysed by **L3**/Pd(dba)_2_ [40].

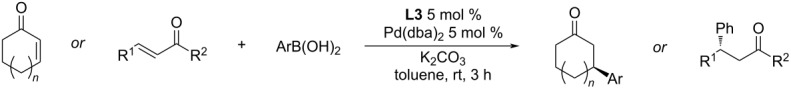					

entry	cyclic substrates		Ar	yield (%)	ee (%)
	*n*				

1	0		Ph	94	54
2	1		Ph	92	76
3	1		4-Me-C_6_H_4_	89	78
4	1		4-MeO-C_6_H_4_	83	76
5	1		4-*t-*Bu-C_6_H_4_	92	79
6	1		4-CF_3_-C_6_H_4_	81	4
7	1		4-F-C_6_H_4_	45	68
8	1		1-naphthyl	80	42
9	2		Ph	90	38

	acyclic substrates				
	R^1^	R^2^			

10	Me	Me	Ph	53	44
11	Me	Et	Ph	62	47
12	iPr	Me	Ph	70	52
13	*n*-C_5_H_11_	Me	Ph	99	42

**Scheme 7:**
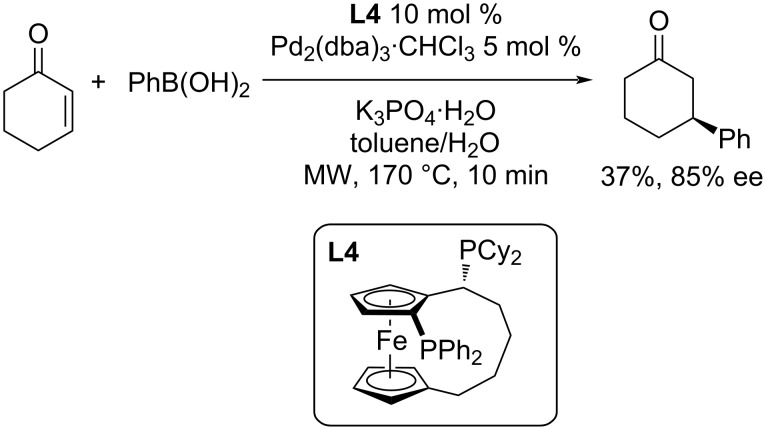
Microwave-assisted addition of phenylboronic acid to 2-cyclohexenone catalysed by **L4**/Pd_2_(dba)_3_·CHCl_3_ [41].

A different approach using microwave irradiation was explored by the group of Toma et al. [41]. After an initial tuning of the reaction conditions of a catalytic system based on Pd(OAc)_2_/2,2’-bipy several optically pure phosphoramidite and diphosphine ligands in combination with Pd_2_(dba)_3_·CHCl_3_ were tested [41]. The obtained yields were within the range of 12–37% with enantioselectivities 12–85% ee. The best level of enantioselectivity was achieved using diphosphine ligand **L4** (Scheme 7). The results in terms of both yield and enantioselectivity were very poor (37%; 85% ee), but the reaction times were very short (Scheme 7) [41].

In 2011, the groups of Hayashi and Chujo studied Pd complexes of diphosphacrown ethers [42]. The macrocyclic Pd complex **PdL5** in combination with AgSbF_6_ or AgOTf was tested for the addition reaction of various arylboronic acids to 2-cyclopentenone. In the case of the addition of phenylboronic acid, high yields and enantioselectivities were achieved (83–92% ee; entries 1–4, Table 11). However, in the case of substituted boronic acids decreased enantioselectivities were observed (72–82% ee; entries 5–8, Table 11) [42].

**Table 11:** Addition of arylboronic acid on 2-cyclopentenone catalysed by **PdL5** [42].

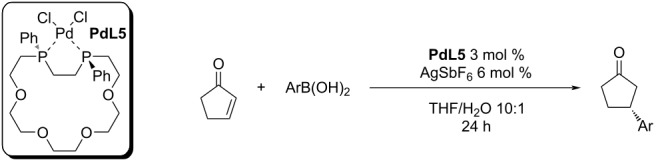				

entry	Ar	temp. (°C)	yield (%)	ee (%)

1	Ph	30	90	85
2	Ph	0	89	87
3	Ph	30	>99^a^	83
4	Ph	0	36^a^	92
5	4-MeO-C_6_H_4_	30	94	82
6	4-CF_3_-C_6_H_4_	30	91	72
7	4-Br-C_6_H_4_	30	95	78
8	2-Me-C_6_H_4_	30	94	72

^a^AgOTf 6 mol % instead of AgSbF_6_.

The most recent systematic study of phosphine-based Pd complexes was done by Wong et al. in 2014. The palladacycle **PdL6** was used in combination with triphenylphosphine and K_3_PO_4_ acting as a base. The highest enantioselectivity of 99% ee of a model addition of phenylboronic acid to 2-cyclohexenone was achieved in dioxane as the solvent, but the yield was only 22%. Therefore, the authors used toluene as the best compromise between yield and enantioselectivity for the next study (Table 12). The addition reaction using the five-membered enone provided the product in moderate yield and enantioselectivity (64%; 50% ee; entry 1, Table 12). On the other hand, the addition of phenylboronic acid to six and seven-membered cycles as well as linear substrates provided the products with high yields (72–97%) and enantioselectivities (78–92% ee; entries 2, 3, 4–12, Table 12). In reactions with substituted arylboronic acids and selected acyclic enones comparable enantioselectivities were observed, while the yields were slightly lower in most cases (56–93% ee, 47–97%; entries 13–21, Table 12) [43].

**Table 12:** Application of dimeric palladacycle **PdL6** in the addition reactions of arylboronic acids to various enones [43].

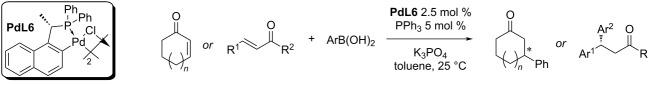					

entry	cyclic substrates		Ar	yield (%)	ee (%)
	*n*				

1	0		Ph	64	50 (*S*)
2	1		Ph	89	92 (*R*)
3	2		Ph	72	87 (*R*)

	acyclic substrates				
	R^1^	R^2^			

4	4-F-C_6_H_4_	Ph	Ph	88	81
5	4-Cl-C_6_H_4_	Ph	Ph	92	78
6	4-Br-C_6_H_4_	Ph	Ph	88	78
7	4-MeO-C_6_H_4_	Ph	Ph	95	81
8	4-Me-C_6_H_4_	Ph	Ph	97	81
9	4-CF_3_-C_6_H_4_	Ph	Ph	92	69
10	2-naphthyl	Ph	Ph	88	85
11	4-Ph-C_6_H_4_	Ph	Ph	85	79
12	3,4-(CH_2_O_2_)-C_6_H_3_	Ph	Ph	95	81
13	Ph	Me	4-Me-C_6_H_4_	63	87
14	Me	Me	Ph	56	93
15	Ph	Ph	2-naphthyl	97	77
16	Ph	Ph	4-F-C_6_H_4_	92	79
17	Ph	Ph	4-Cl-C_6_H_4_	56	82
18	Ph	Ph	4-Br-C_6_H_4_	88	56
19	Ph	Ph	4-Me-C_6_H_4_	89	69
20	Ph	Ph	4-MeO-C_6_H_4_	83	85
21	Ph	Ph	4-CF_3_-C_6_H_4_	47	80

**Scheme 8:**
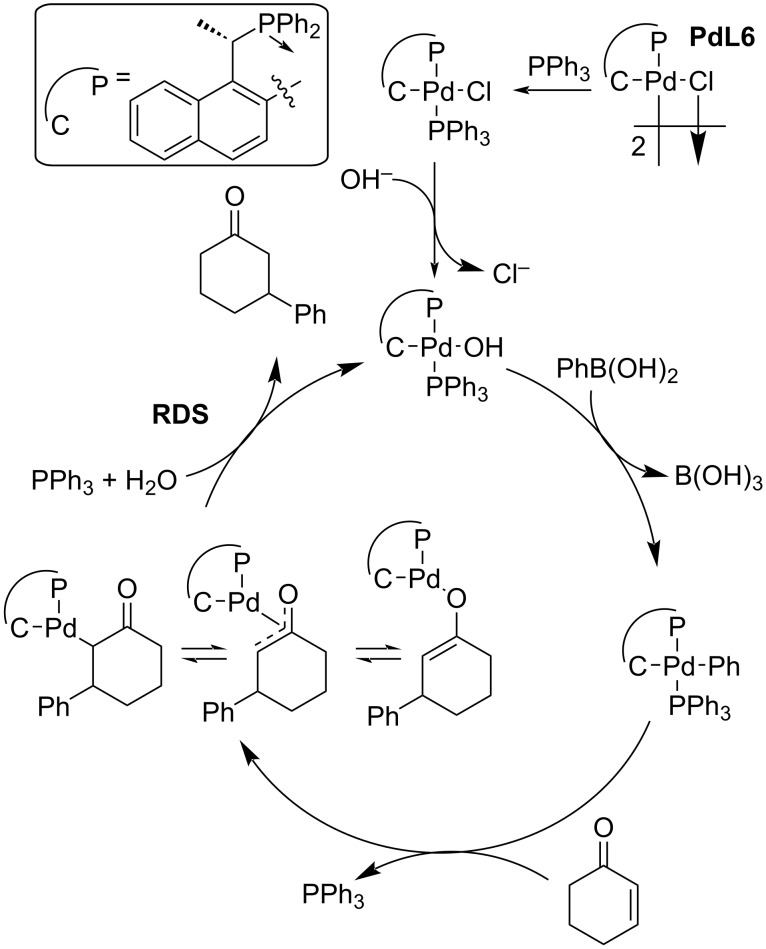
Plausible catalytic cycle of the addition of phenylboronic acid to 2-cyclohexenone catalysed by palladacycle **PdL6** [43].

Furthermore, the authors proposed a catalytic cycle (Scheme 8) [43] and stated that the rate-determining step (RDS) was the protonolysis of the *O*-bound enolate in the presence of PPh_3_ that leads to the regeneration of the catalytically active hydroxopalladium species and the addition product (Scheme 8) [43]. The presence of PPh_3_ ensures the preference of hydrolysis instead of a β-hydride elimination, which would lead to an oxidative Heck-type product. The authors stated that as a result of the coordination with PPh_3_, there is a steric hindrance disfavouring the β-hydride elimination [43].

### Catalytic systems based on NHC ligands

Historically, the second type of ligands used were *N*-heterocyclic carbenes (NHC). The first use was reported in a work Shi and co-workers in 2008 who studied the addition of arylboronic acids to 2-cyclohexenone catalysed by Pd complexes of axially chiral NHC carbenes with two other weakly coordinating ligands [44–45]. The complexes with acetates (**PdL7a**), trifluoroacetates (**PdL7b**), and diaquo complex (**PdL7c**) provided similar results in the reactions with simple enones (Table 13). The authors discussed the need for the presence of KOH as a base [44–45]. Without the base the reaction did not give any product.

**Table 13:** Addition reaction of boronic acids to 2-cyclohexenone, catalysed by Pd-NHC complexes **PdL7a–c** [44–45].

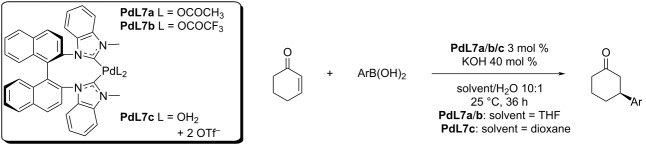				

entry	Ar	catalyst	yield (%)	ee (%)

1	Ph	**PdL7a**	95	93
2	Ph	**PdL7b**	97	96
3	Ph	**PdL7c**	98	95
4	3-Me-C_6_H_4_	**PdL7b**	97	97
5	3-Me-C_6_H_4_	**PdL7c**	95	92
6	4-Me-C_6_H_4_	**PdL7b**	89	92
7	4-Me-C_6_H_4_	**PdL7c**	83	90
8	3-MeO-C_6_H_4_	**PdL7a**	92	94
9	3-MeO-C_6_H_4_	**PdL7b**	90	97
10	3-MeO-C_6_H_4_	**PdL7c**	90	97
11	4-MeO-C_6_H_4_	**PdL7b**	82	84
12	2-naphtyl	**PdL7a**	98	96
13	2-naphtyl	**PdL7b**	99	97
14	2-naphtyl	**PdL7c**	99	96
15	4-Ph-C_6_H_4_	**PdL7b**	97	93
16	3-Cl-C_6_H_4_	**PdL7b**	78	88
17	3-Cl-C_6_H_4_	**PdL7c**	78	86
18	3,5-diMe-C_6_H_3_	**PdL7b**	90	92
19	3,5-diMe-C_6_H_3_	**PdL7c**	95	88

The broadening of the reaction scope showed that the catalysts were also suitable for reactions with seven-membered cyclic enones. However, the effectiveness was decreased in the case of five-membered rings or heterocyclic six-membered rings as the substrates (Table 14) [44].

**Table 14:** Addition reaction of arylboronic acids to different enones catalysed by Pd-NHC complexes **PdL7a–c** [44–45].

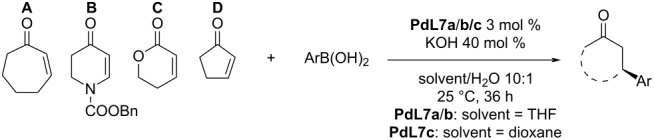					

entry	substrate	Ar	catalyst	yield (%)	ee (%)

1	**A**	Ph	**PdL7a**	85	94
2	**A**	Ph	**PdL7b**	88	91
3	**A**	Ph	**PdL7c**	85	94
4	**A**	4-Me-C_6_H_4_	**PdL7b**	90	91
5	**A**	3-MeO-C_6_H_4_	**PdL7b**	86	96
6	**A**	3-MeO-C_6_H_4_	**PdL7c**	84	96
7	**A**	2-naphthyl	**PdL7a**	84	96
8	**A**	2-naphthyl	**PdL7b**	99	97
9	**A**	2-naphthyl	**PdL7c**	93	94
10	**B**	Ph	**PdL7b**	53^a^	81
11	**C**	Ph	**PdL7b**	62^a^	38
12	**D**	Ph	**PdL7b**	58	32

^a^reaction temperature 50 °C.

**Scheme 9:**
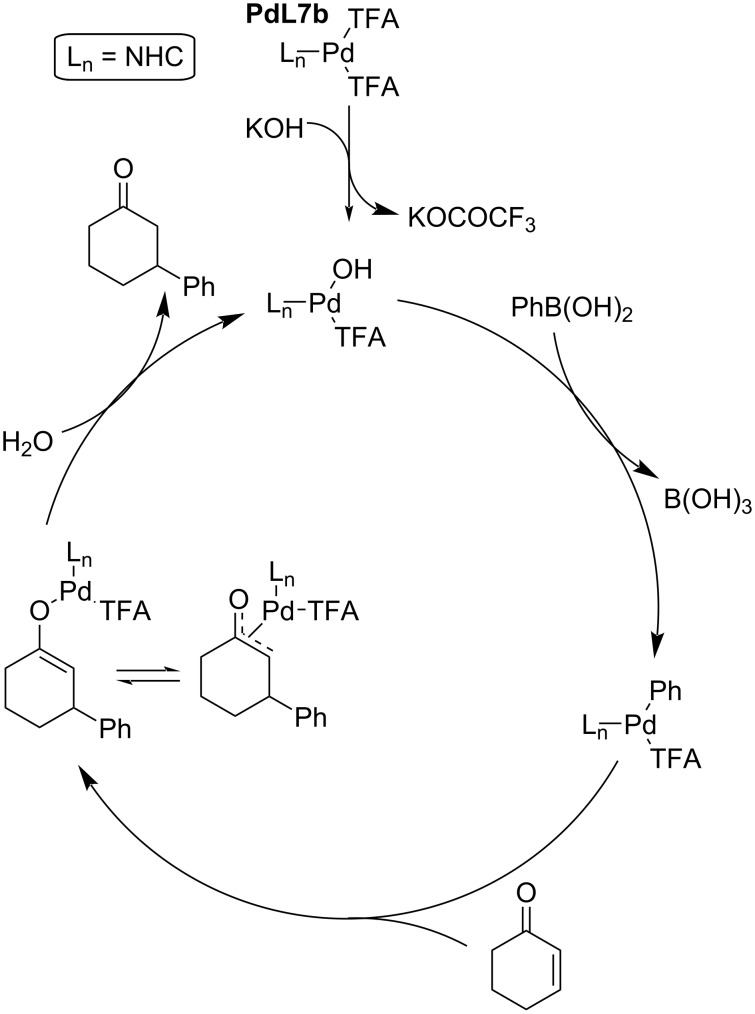
Proposed catalytic cycle for the addition of phenylboronic acids to 2-cyclohexenone catalysed by Pd-NHC complex **PdL7b** [44].

The unsatisfactory result obtained for substrate **B** (entry 10, Table 14) was overcome in the next work that focused on the optimisation of the reaction conditions for the addition of arylboronic acids to substituted dihydropyridones. Under the optimised conditions, 1,4-dioxane was used instead of THF as a solvent. The obtained results for the additions of various boronic acids to a series of alkyl 4-oxo-3,4-dihydropyridine-1(2*H*)-carboxylates were excellent in terms of both conversion (72–96%) and enantioselectivities (87–99% ee; Table 15) [45]. In addition, the authors proposed a catalytic cycle for this reaction (Scheme 9).

**Table 15:** Addition reaction of arylboronic acids to various 4-oxo-3,4-dihydropyridine-1(2*H*)-carboxylates catalysed by Pd-NHC complexes **PdL7a–c** [45].

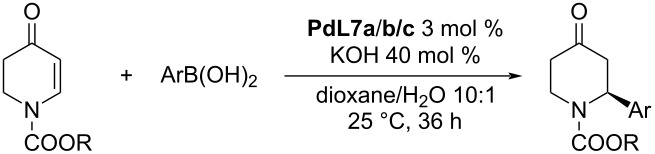					

entry	R	Ar	catalyst	yield (%)	ee (%)

1	Bn	Ph	**PdL7a**	86	99
2	Bn	Ph	**PdL7b**	88	>99
3	Bn	Ph	**PdL7c**	88	>99
4	Bn	4-Me-C_6_H_4_	**PdL7b**	85	96
5	Bn	4-Me-C_6_H_4_	**PdL7c**	82	95
6	Bn	3-Me-C_6_H_4_	**PdL7b**	80	95
7	Bn	3-Me-C_6_H_4_	**PdL7c**	80	98
8	Bn	4-MeO-C_6_H_4_	**PdL7b**	78	>99
9	Bn	4-MeO-C_6_H_4_	**PdL7c**	82	>99
10	Bn	3-MeO-C_6_H_4_	**PdL7b**	76	99
11	Bn	3-MeO-C_6_H_4_	**PdL7c**	72	90
12	Bn	2-naphthyl	**PdL7b**	85	98
13	Bn	2-naphthyl	**PdL7c**	86	97
14	Bn	4-Ph-C_6_H_4_	**PdL7b**	94	97
15	Bn	4-Ph-C_6_H_4_	**PdL7c**	96	98
16	Et	Ph	**PdL7b**	92	87
17	Et	Ph	**PdL7c**	90	98
18	Et	2-naphthyl	**PdL7b**	85	97
19	Et	4-Ph-C_6_H_4_	**PdL7b**	95	97
20	*t-*Bu	Ph	**PdL7b**	82	99
21	*t-*Bu	Ph	**PdL7c**	80	98
22	*t-*Bu	2-naphthyl	**PdL7b**	80	97
23	*t-*Bu	4-Ph-C_6_H_4_	**PdL7b**	95	>99

In 2013, the most recent NHC-Pd based system has been developed by Mullick et al. who used ligands derived from *trans*-9,10-dihydro-9,10-ethanoanthracene-11,12-diyl (DEA) and *trans*-9,10-dihydro-9,10-ethanoanthracene-11,12-diylmethanediyl (DEAM) in form of Pd-bisNHC complexes [46]. The catalysts were prepared in situ and tested for the addition reaction of various boronic acids to five and six-membered enones (Table 16). The results were unsatisfactory in terms of yield and enantioselectivity (24–98%; 30–51% ee) and most of the studied combinations gave no product or the authors were not able to determine the enantioselectivity. A selection of some interesting results is summarised in Table 16 [46].

**Table 16:** Addition reactions of boronic acids to five and six-membered enones catalysed by in situ-prepared Pd-bisNHC complex **PdL8** [46].

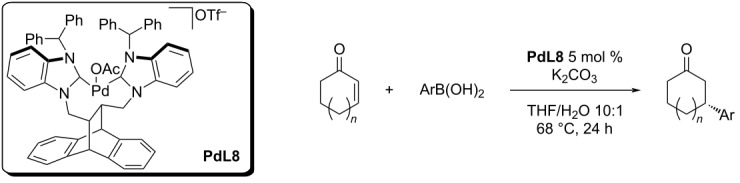				

entry	*n*	Ar	yield (%)	ee (%)

1	0	2-Me-C_6_H_4_	36	50
2	0	2-MeO-C_6_H_4_	35	51
3	0	4-MeO-C_6_H_4_	30	35
4	0	1-naphthyl	24	30
5	1	Ph	98	51
6	1	2-Me-C_6_H_4_	62	33
7	1	1-naphthyl	48	30

### Catalytic systems based on pyridine-oxazolines ligands

Currently, the most studied ligand class is focused on pyridine-oxazolines (PyOx). The first report for the use of this type of ligand for the asymmetric addition of arylboronic acids to cyclic enones was published by the Stoltz group in 2011 [47]. The most efficient catalytic system was identified as a combination of (*S*)-*t-*Bu-PyOx (**L9**) with Pd(TFA)_2_ (Table 17). This system exhibited a remarkable tolerance for water and air. It was demonstrated by the addition of 10 equiv of water into the reaction mixture that caused only a very small decrease of the enantioselectivity from 93% ee to 91% ee (entries 1 and 2, Table 17). Additional deuteration experiments demonstrated that water acted as a proton source in the catalytic cycle [48]. Furthermore, only a very low conversion was achieved without water, especially in large-scale experiments. Proton sources other than water were tested too. The use of MeOH or *t-*BuOH resulted in a 10 to 15% decrease of enantioselectivity and 2,2,2-trifluoroethanol (TFE) had only a minimal impact on the enantioselectivity. The benefit of using TFE instead of water was its miscibility with the reaction medium (DCE) [48].

A series of different arylboronic acids was tested for the addition reaction to 3-methyl-2-cyclohexenone (Table 17). Electron-poor arylboronic acids gave generally better enantioselectivities than electron-rich arylboronic acids [47,49].

**Table 17:** Addition reaction of arylboronic acids to 3-methyl-2-cyclohexenone catalysed by **L9**/Pd(TFA)_2_ [47,49].

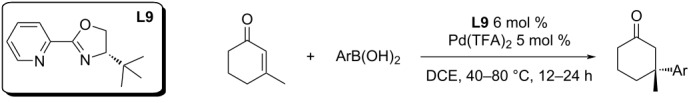					

entry	Ar	temp. (°C)	time (h)	yield (%)	ee (%)

1	Ph	60	12	99	93
2	Ph	60	12	99	91^a^
3	4-Me-C_6_H_4_	60	12	99	87
4	4-Et-C_6_H_4_	60	12	90	85
5	4-MeO-C_6_H_4_	40	24	58	69
6	4-BnO-C_6_H_4_	60	18	96	74
7	4-TBSO-C_6_H_4_	40	24	52	82
8	4-Ac-C_6_H_4_	60	18	99	96
9	4-Cl-C_6_H_4_	60	12	94	95
10	4-F-C_6_H_4_	80	12	84	92
11	2-F-C_6_H_4_	60	12	32	77
12	4-CF_3_-C_6_H_4_	60	12	99	96
13	3-Me-C_6_H_4_	60	24	99	91
14	3-Cl-C_6_H_4_	60	18	55	96
15	3-Br-C_6_H_4_	60	24	44	85
16	3-MeOOC-C_6_H_4_	60	24	91	95
17	3-NO_2_-C_6_H_4_	60	18	40	92

^a^Addition of 10 equiv of water.

Different enone substrates varying in ring size and substitution in the 3-position were also tested. The products were usually obtained with a high degree of enantioselectivity in good yields (up to 96%; up to 93% ee; Table 18) [47,49].

**Table 18:** Addition reactions of phenylboronic acid to various 3-substituted enones catalysed by **L9**/Pd(TFA)_2_ [47,49].

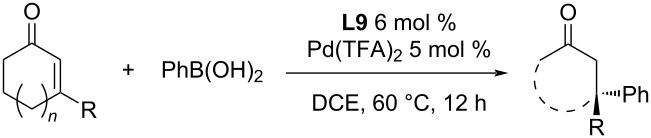				

entry	*n*	R	yield (%)	ee (%)

1	0	Me	84	91
2	2	Me	85	93
3	1	Et	96	92
4	1	*n*-Bu	95	91
5	1	Bn	74	91
6	1	Cy	86	85
8	1	iPr	86	79
7	1	cyclopropyl	68	88
9	1	(CH_2_)_3_OBn	65	91

An interesting finding was the effect of non-coordinating hexafluorophosphate anions. The addition of 30 mol % NH_4_PF_6_ increased the catalytic activity and allowed to run the reaction at a lower temperature [48]. This can be very useful for substrates that can react with traces of Pd(0) that are formed by minor side reactions. The authors suspected that hexafluorophosphate anions stabilize the cationic Pd species and result in its increased solubility. The impact of the addition of 30 mol % NH_4_PF_6_ caused that the product yield was almost doubled even when the temperature was 20 °C lower (Table 19) [48], while there was only a minimal to no effect on the enantioselectivity (Table 19). Scale-up to a gram-scale was possible, without a major loss of either yield or enantioselectivity (entry 7, Table 19) [50].

**Table 19:** Effect of ammonium hexafluorophosphate as additive on the addition reactions of arylboronic acids to 3-methyl-2-cyclohexanone catalysed by **L9**/Pd(TFA)_2_ [48,50].

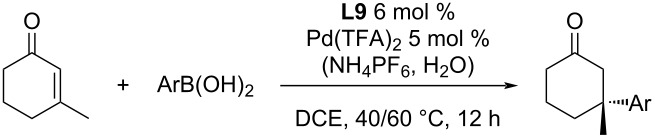						

	60 °C, without additive			40 °C, 30 mol % NH_4_PF_6_, 5 equiv H_2_O		
Ar	entry	yield (%)	ee (%)	entry	yield (%)	ee (%)

3-Cl-C_6_H_4_	1	55	97	6	96	96
4-Cl-C_6_H_4_	2	94	95	7^a^	87–91	93
3-Br-C_6_H_4_	3	44	86	8	84	84
3-NO_2_-C_6_H_4_	4	40	92	9	81	91
2-F-C_6_H_4_	5	32	77	10	70	77

^a^Reaction performed at a 35 mmol scale [50].

The substrate scope was further expanded with addition reactions of arylboronic acids to 3-acetyl-2-cyclohexenone. The products were isolated in moderate to good yields and excellent enantioselectivities (57–92%; 90–95% ee). Furthermore, no 2-arylated products have been detected (Table 20) [49].

**Table 20:** Addition reactions of arylboronic acids to 3-acetyl-2-cyclohexanone catalysed by **L9**/Pd(TFA)_2_ [49].

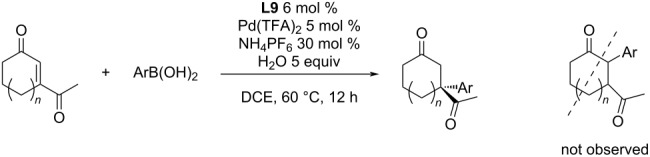				

entry	*n*	Ar	yield (%)	ee (%)

1	1	4-Cl-C_6_H_4_	85	96
2	1	4-F-C_6_H_4_	92	90
3	1	3-Me-C_6_H_4_	66	92
4	1	3-(CF_3_CONH)-4-Me-C_6_H_3_	73	91
5	0	Ph	72	93
6	0	3-Me-C_6_H_4_	72	90
7	0	4-F-C_6_H_4_	57	92

Next, the substrate scope was further expanded with the addition reactions of *N*-protected aminophenylboronic acids. The best results in terms of enantioselectivity were achieved when trifluoroacetyl was used as the *N*-protecting group (Table 21) [49].

**Table 21:** Addition reactions of *N*-protected aminophenylboronic acids to 3-methyl-2-cyclohexanone catalysed by **L9**/Pd(TFA)_2_ [49].

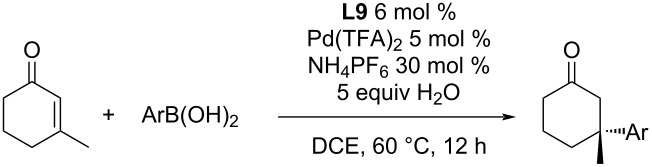			

entry	Ar	yield (%)	ee (%)

1	4-(Cbz-NH)-C_6_H_4_	45	76
2	4-(Boc-NH)-C_6_H_4_	72	78
3	4-(CF_3_CONH)-C_6_H_4_	98	89
4	4-(CF_3_CONH)-3-Me-OC_6_H_3_	75	88
5	4-(CF_3_CONH)-3,5-diMeO-C_6_H_2_	93	90
6	3-(CF_3_CONH)-C_6_H_4_	60	92
7	3-(CF_3_CONH)-4-MeO-C_6_H_3_	77	88

In other experiments, Stoltz and co-workers showed the ineffectiveness of the **L9**/Pd(TFA)_2_ catalytic system for the addition of phenylboronic acid to nonsubstituted 2-cyclohexenone, yielding the product with very low enantioselectivity (18%; entry 1, Table 22). Furthermore, the addition reaction to a 6,6,3-trimethylated substrate gave the product in only very low yield (9%), but with high enantioselectivity (90% ee; entry 3, Table 22) [48]. The application of the catalytic system in the addition reaction to an unsaturated lactone yielded the product with both low yield and enantioselectivity (49%; 57% ee; entry 4, Table 22) [48]. Finally, the catalytic system failed in the addition reaction with 2-methylchromone and did not yield the expected product, however, it proved to be highly effective for the addition reaction to unsubstituted chromone (91%; 94% ee; entry 5, Table 22) [51].

**Table 22:** Addition reaction of phenylboronic acid to various enones, lactones, and chromones catalysed by **L9**/Pd(TFA)_2_ [48,51].

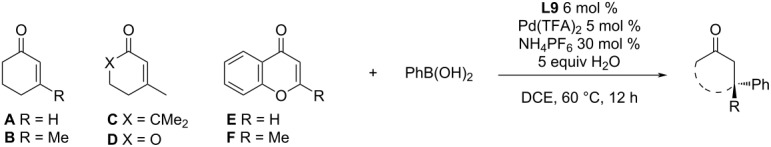			

entry	substrate	yield (%)	ee (%)

1	**A**	87 (no NH_4_PF_6_)	18
2	**B**	99	93
3	**C**	9^a^	90
4	**D**	49^a^	57
5	**E**	91	94
6	**F**	0	–

^a^Reaction temperature 40 °C.

According to these findings, Stoltz and co-workers tested the catalytic system with a library of different chromones for the addition of various boronic acids. The substituted flavanones were obtained with moderate to good yields (36–96%) and usually very high levels of enantioselectivity (up to 98% ee; entries 1–29, Table 23) [51]. Also, the addition reaction to the structurally similar *N*-Cbz-4-quinolone was tested, resulting in the corresponding products with only low to moderate yields (31–65%) and moderate to good enantioselectivities (40–89% ee; entries 30–38, Table 23) [51].

**Table 23:** Addition reactions of arylboronic acids to substituted chromones and *N*-Cbz-4-quinolones catalysed by **L9**/Pd(TFA)_2_ [51].

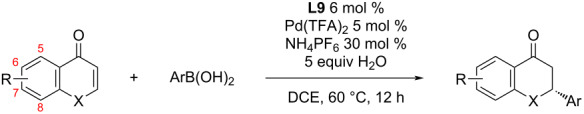					

entry	X	R	Ar	yield (%)	ee (%)

1	O	H	Ph	91	94
2	O	H	2-F-C_6_H_4_	50	76
3	O	H	3-Me-C_6_H_4_	66	90
4	O	H	3-MeOOC-MeC_6_H_4_	72	93
5	O	H	3-Br-C_6_H_4_	40	89
6	O	H	3-(CF_3_CONH)-C_6_H_4_	77	98
7	O	H	3-Cl-C_6_H_4_	52	94
8	O	H	4-Me-C_6_H_4_	64	94
9	O	H	4-Et-C_6_H_4_	36	85
10	O	H	4-F-C_6_H_4_	51	90
11	O	H	3,5-diMeO-C_6_H_3_	69	95
12	O	H	dibenzofuran-4-yl	64	77
13	O	6-Ac-5,7-diMe	Ph	98	90
14	O	6-Ac-5,7-diMe	3-Me-C_6_H_4_	76	88
15	O	6-Ac-5,7-diMe	4-Et-C_6_H_4_	45	86
16	O	6-Ac-5,7-diMe	Ph	79	95
17	O	6-Ac-5,7-diMe	3-Me-C_6_H_4_	84	86
18	O	6-Ac-5,7-diMe	3-Br-C_6_H_4_	65	95
19	O	6-Ac-5,7-diMe	4-F-C_6_H_4_	68	91
20	O	6-Ac-5,7-diMe	3-MeOOC-C_6_H_4_	90	86
21	O	6-Ac-5,7-diMe	dibenzofuran-4-yl	70	83
22	O	5,7-diMe	Ph	84	93
23	O	5,7-diMe	4-(CF_3_CONH)-3-MeO-C_6_H_3_	80	95
24	O	7-OAc	Ph	77	92
25	O	7-OH	Ph	77	93
26	O	7-OH	3-Me-C_6_H_4_	66	90
27	O	7-OH	4-F-C_6_H_4_	50	93
28	O	7-MeO	Ph	96	94
29	O	7-MeO	3-MeOOC-C_6_H_4_	81	96
30	NCbz	H	Ph	50	80
31	NCbz	H	3-(CF_3_CONH)-4-Me-C_6_H_3_	45	85
32	NCbz	H	3-Me-C_6_H_4_	51	85
33	NCbz	H	3,5-diMeO-C_6_H_3_	50	85
34	NCbz	H	3-MeOOC-C_6_H_4_	34	60
35	NCbz	H	4-F-C_6_H_4_	65	89
36	NCbz	H	4-Me-C_6_H_4_	45	67
37	NCbz	H	4-MeO-C_6_H_4_	36	54
38	NCbz	H	dibenzofuran-4-yl	31	40

**Scheme 10:**
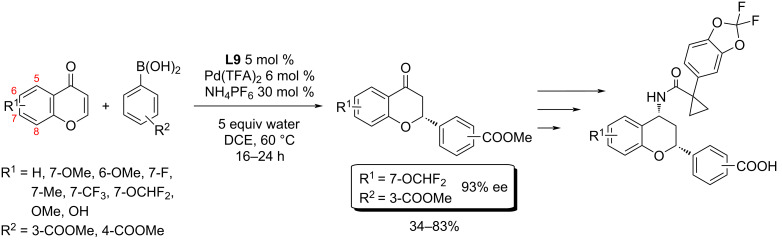
Usage of addition reactions of boronic acids to various chromones in the syntheses of potentially active substances in medicinal chemistry [5].

In 2018, Wang et al. applied the optimised reaction conditions for the synthesis of various compounds that could be potentially usable for the treatment of cystic fibrosis (Scheme 10) [5].

**Scheme 11:**
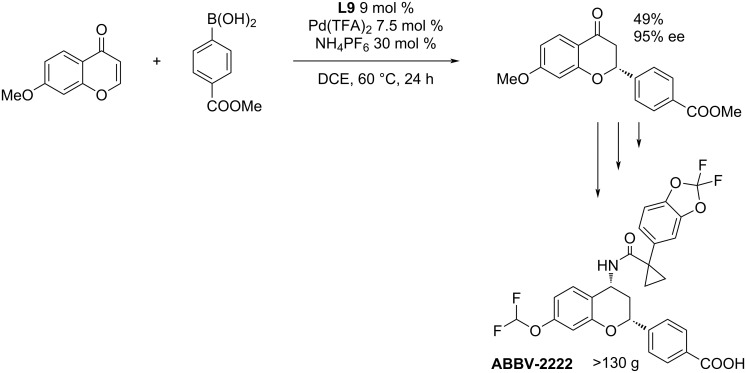
Multigram-scale synthesis of **ABBV-2222** [6].

The large-scale synthesis (>130 g) of the most successful hit was later published by Greszler et al. (Scheme 11) [6].

In 2019, another expansion of the substrate scope for the synthesis of substituted flavanones was done by Liu et al. (Table 24). The prepared flavanones were further tested for their cancerostatic activity [7].

**Table 24:** Addition reactions of arylboronic acids to substituted chromones catalysed by **L9**/Pd(TFA)_2_ [7].

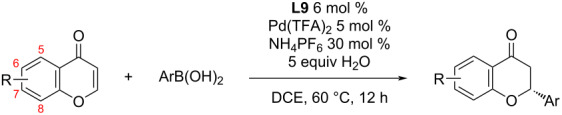				

entry	R	Ar	yield (%)	ee (%)

1	H	Ph	88	94
2	H	3,4-diMeO-C_6_H_3_	58	89
3	H	4-MeO-C_6_H_4_	68	95
4	H	3-MeO-C_6_H_4_	62	86
5	H	3,4,5-triOMe-C_6_H_2_	70	92
6	H	piperonyl	59	89
7	H	4-NO_2_-C_6_H_4_	52	77
8	H	4-Me-C_6_H_4_	63	91
9	H	3-Me-C_6_H_4_	70	83
10	H	4-Cl-C_6_H_4_	50	96
11	H	3-Cl-C_6_H_4_	58	92
12	H	4-Br-C_6_H_4_	49	86
13	H	4-F-C_6_H_4_	46	75
14	H	1-naphthyl	59	78
15	H	2-furyl	55	74
16	H	thiophene-2-yl	45	87
17	H	4-Me_2_N-C_6_H_4_	43	83
18	H	4-Et-C_6_H_4_	58	77
19	H	4-MeS-C_6_H_4_	72	90
20	H	4-*t-*Bu-C_6_H_4_	66	91
21	7-MeO	4-MeO-C_6_H_4_	76	90
22	7-OBn	4-MeO-C_6_H_4_	83	74
23	7-Br	4-MeO-C_6_H_4_	70	93
24	7-F	4-MeO-C_6_H_4_	52	66
25	7-Me	4-MeO-C_6_H_4_	80	82
26	6-Cl-7-Me	4-MeO-C_6_H_4_	68	79
27	7-Cl-6-Me	4-MeO-C_6_H_4_	57	70
28	6-Cl	4-MeO-C_6_H_4_	70	95
29	6-Br	4-MeO-C_6_H_4_	59	76
30	6-F	4-MeO-C_6_H_4_	60	80
31	6-MeO	4-MeO-C_6_H_4_	87	94
32	6-Me	4-MeO-C_6_H_4_	44	79
33	6-NO_2_	4-MeO-C_6_H_4_	67	95
34	6,7-diMeO	4-MeO-C_6_H_4_	48	85
35	5-MeO	4-MeO-C_6_H_4_	75	94
36	5,7-diOMe	4-MeO-C_6_H_4_	65	89
37	6,8-diCl	4-MeO-C_6_H_4_	83	93
38	benzo[*f*]	4-MeO-C_6_H_4_	88	77
39	5,7-bis(MEM)	4-MeO-C_6_H_4_	74	88
40	7-OCH_2_OMe	4-MeO-C_6_H_4_	47	81
41	5,7-diOH	4-MeO-C_6_H_4_	86	–
42	5-OH	4-MeO-C_6_H_4_	89	–

**Scheme 12:**
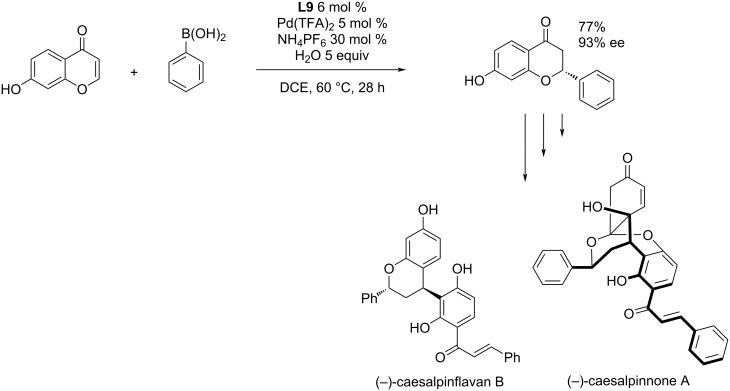
Application of the asymmetric addition of phenylboronic acid to a chromone derivative for the total syntheses of the natural products (−)-caesalpinnone A and (−)-caesalpinflavan B [9].

In 2019, Timmerman et al. applied the asymmetric addition of phenylboronic acid to a chromone derivative for the total syntheses of (−)-caesalpinnone A and (−)-caesalpinflavan B (Scheme 12) [9].

**Scheme 13:**
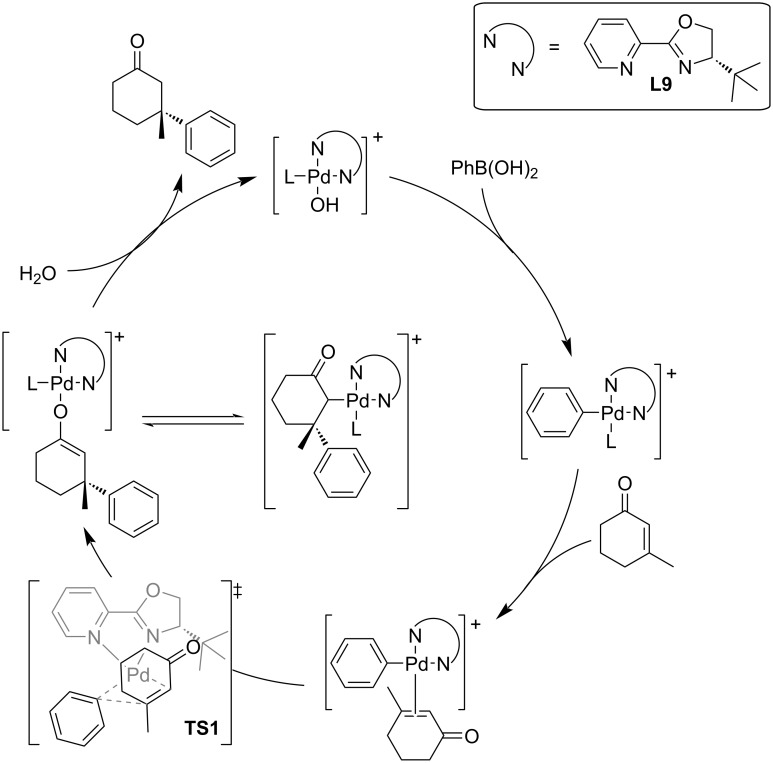
Plausible catalytic cycle for the addition of phenylboronic acid to 3-methyl-2-cyclohexenone catalysed by **L9**/Pd(TFA)_2_ [48–49].

Mechanistic studies of this catalytic system were also made by Stoltz’s group. A linear relationship between the ee of the catalyst and the product has been found [48]. That means that the catalytically relevant species is monomeric Pd–PyOx. This was further supported by a mass spectrometric study [52]. The catalytic cycle was also suggested in accordance with DFT calculations and mechanistic studies (Scheme 13) [48–49]. The key step for both, the enantioselectivity and turnover, is the migratory insertion via **TS1** (Scheme 13). The stereochemistry is controlled mainly by the hydrogen repulsion of the methylene group neighbouring the keto group of the enone with the *t-*Bu group of the ligand **L9**.

**Scheme 14:**
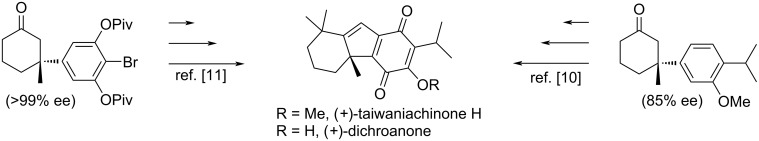
Total syntheses of naturally occurring terpenoids [10–11].

Another interesting example for the application of this reaction in the preparation of precursors of natural molecules was reported by Li et al. in 2014. They presented the synthesis of terpenoid precursors ((+)-taiwaniaquinone H and (+)-dichroanone) [10] starting from 3-methyl-2-cyclohexenone using the **L9**/Pd(TFA)_2_ catalytic system. The precursors were prepared in good yields (42–98%) with high enantioselectivities (85–99% ee; entry 1; Table 25) and used in the total synthesis of terpenoids (Scheme 14) [10].

**Table 25:** Addition of various highly functionalized arylboronic acids to 3-methylcyclohexanone for the synthesis of terpenoids [10–11].

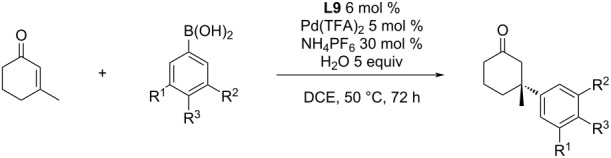					

entry	R^1^	R^2^	R^3^	yield (%)	ee (%)

1	MeO	H	iPr	89^a^	85
2	MeO	MeO	iPr	trace	–
3	PivO	PivO	Ac	93	94
4	PivO	PivO	I	42	92
5	PivO	PivO	Br	98	>99
6	PivO	PivO	Cl	94	>99

^a^Reaction performed at 60 °C for 48 h.

In the same year, these terpenoids were also prepared by the Stoltz group [11]. Arylboronic acids bearing the appropriate functional groups were identified and the addition reactions to 3-methyl-2-cyclohexenone were studied (entries 2–6, Table 25) [11]. The product, which was obtained in an almost quantitative yield and practically maximal possible enantioselectivity (entry 5 in Table 25), was subsequently converted to suitable intermediates for the synthesis of naturally occurring terpenoids (Scheme 14) [11].

**Scheme 15:**
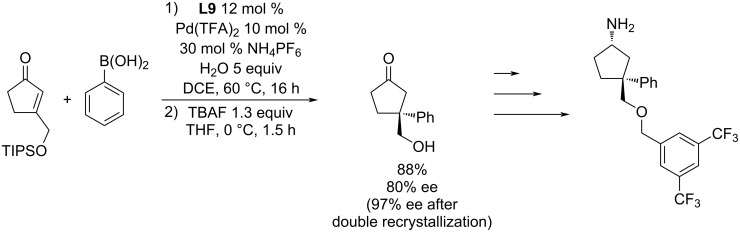
Use of the **L9**/Pd(TFA)_2_ catalytic system for the synthesis of intermediates of biologically active compounds [4].

Another possible use of this catalytic system was demonstrated by the groups of Lautens and Hashmi [4]. The starting enone, prepared by the Au(I)-catalysed Rautenstrauch rearrangement, was subjected to the addition reaction with phenylboronic acid (Scheme 15). Without isolation of the intermediate, the protecting group was removed and the product was obtained in 88% yield and 80% ee. The enantiomeric excess of the obtained (*S*)-3-(hydroxymethyl)-3-phenyl-2-cyclopentanone could be increased by double recrystallization to up to 97% ee (Scheme 15) [4].

**Scheme 16:**
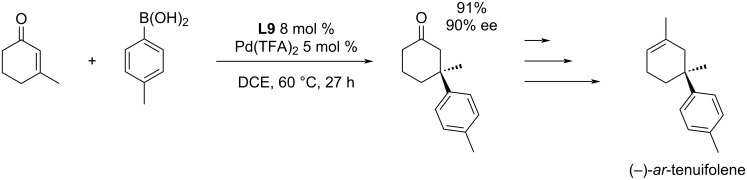
Usage of a Michael addition catalysed by **L9**/Pd(TFA)_2_ in the total synthesis of (–)-*ar*-tenuifolene [12].

The catalytic system **L9**/Pd(TFA)_2_ was further used in the work published in 2020 by Bisai et al. for the addition of 4-tolylboronic acid to 3-methyl-2-cyclohexenone in the total synthesis of the aromatic sesquiterpene (−)-*ar*-tenuifolene (Scheme 16) [12].

**Scheme 17:**
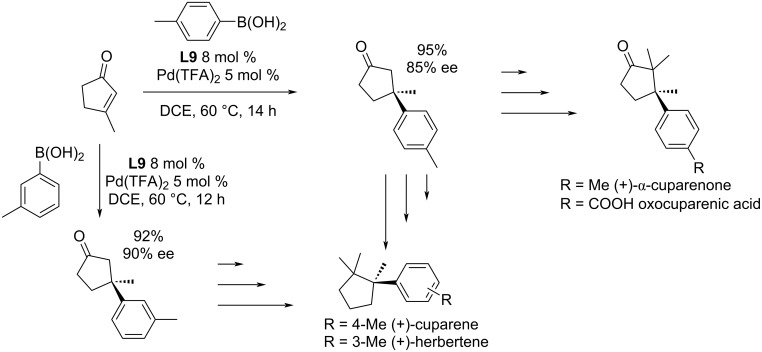
Synthesis of terpenoids by Michael addition to 3-methyl-2-cyclopentenone [13].

Later in 2020, Bisai et al. published the application of the **L9**/Pd(TFA)_2_ catalytic system for the preparation of the enantiomers of other sesquiterpenoids by the addition reactions of tolylboronic acids to 3-methyl-2-cyclopentenone (Scheme 17) [13].

**Scheme 18:**
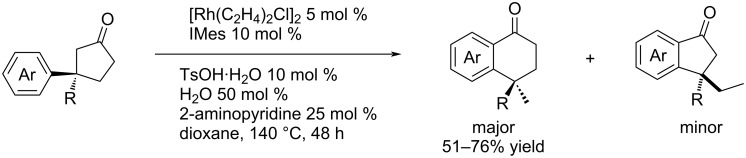
Rh-catalysed isomerisation of 3-alkyl-3-arylcyclopentanones to 1-tetralones [53].

Also in 2020, Ochi et al. expanded the synthetic usability of 3-alkyl-3-arylcyclopentanones by developing a method for their Rh-catalysed isomerisation to 1-tetralones with >99% stereoretention (Scheme 18) [53].

To obtain the starting material for the transformation (Scheme 18), the authors have described the addition of arylboronic acids to 3-substituted-2-cyclopentenones (Table 26) either by using Stoltz’s catalytic system **L9**/Pd(TFA)_2_ or by its simple modification (temperature, catalyst loading) combined with the iterative addition of boronic acids (1 equiv immediately and 1 equiv after 3 hours) [49].

**Table 26:** Addition reactions of arylboronic acids to 3-alkyl-2-cyclopentenones catalysed by **L9**/Pd(TFA)_2_ [53].

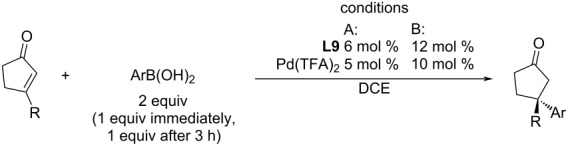						

entry	R	Ar	temp. (°C)	conditions	yield (%)	ee (%)

1	Et	Ph	25	A	95	94
2	Et	4-Me-C_6_H_4_	25	B	67	91
3	Et	4-MeO-C_6_H_4_	25	A	49	84
4	Et	4-MeO-C_6_H_4_	60	B	63	74
5	Et	4-Bu-C_6_H_4_	60	B	91	84
6	Et	4-Cl-C_6_H_4_	60	B	78	93
7	Et	4-F-C_6_H_4_	60	B	84	92
8	Et	4-CF_3_-C_6_H_4_	25	A	6	95
9	Et	4-CF_3_-C_6_H_4_	60	B	99	94
10	Et	4-MeOOC-C_6_H_4_	60	B	99	94
11	Et	3-Me-C_6_H_4_	60	B	97	91
12	Bu	Ph	25	A	82	96
13	Cy	Ph	60	A	91	96
14	(CH_2_)_2_COOMe	Ph	60	A	86	97

**Scheme 19:**
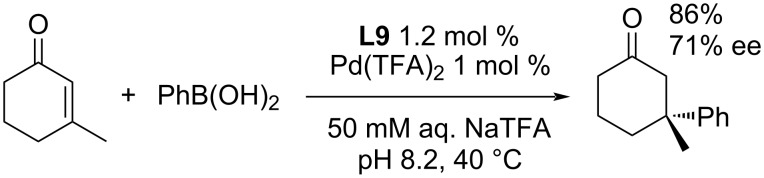
Addition reaction of phenylboronic acid to 3-methyl-2-cyclohexenone catalysed by **L9**/Pd(TFA)_2_ in water [54].

Following Stoltz's works [11,27,47–49,51–52], Stanley et al. published the first example for the formation of all-carbon quaternary stereocentres, in an aqueous medium (Scheme 19) [54] by the addition of phenylboronic acid to 3-methyl-2-cyclohexenone using the **L9**/Pd(TFA)_2_ catalytic system. Compared to the reaction in DCE (93% yield, 92% ee,) [47], a slightly lower yield and significantly lower enantioselectivity were obtained in water as the solvent (86% yield, 71% ee, Scheme 19) [54].

Significant successes of the Stanley group were achieved in the subsequent study of the as yet unexplored asymmetric addition of arylboronic acids to 3-aryl-2-cyclohexenones, where double benzyl quaternary stereogenic centres were formed [55]. The initial studies showed the formation of significant amounts of protodeborylation products, small amounts of boronic acid homocoupling products, and the corresponding phenols as boronic acid oxidation products. To optimise the yields, the amount of the boronic acid was increased to 3 equiv, which was added gradually (1 equiv every 3 hours) [55]. The authors presented interesting results and expanded the range of compounds that could be prepared by this methodology. The obtained results were excellent both in terms of enantioselectivity (up to 91% ee) and conversion (92%; Table 27) [55].

**Table 27:** Addition reactions of arylboronic acids to 3-aryl-2-cyclohexenones catalysed by **L9**/Pd(TFA)_2_ [55].

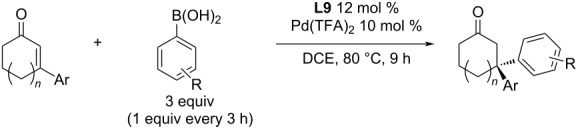					

entry	*n*	Ar	R	yield (%)	ee (%)
1^a^	1	4-MeO-C_6_H_4_	4-Me	83 (81)^b,c^	89 (87)^b,c^
2^a^	1	4-MeO-C_6_H_4_	H	70^c^	87^c^
3	1	4-MeO-C_6_H_4_	4-Ph	92	90
4	1	4-MeO-C_6_H_4_	4-Cl	55	83
5	1	4-MeO-C_6_H_4_	4-F	49	91
6	1	4-MeO-C_6_H_4_	4-COOMe	39	87
7	1	4-MeO-C_6_H_4_	4-CF_3_	38	82
8	1	4-MeO-C_6_H_4_	3-Me	88	90
9	1	4-MeO-C_6_H_4_	3-MeO	60	90
10	1	4-MeO-C_6_H_4_	3-Cl	35	85
11	1	4-MeO-C_6_H_4_	3-F	18	84
12	1	4-MeO-C_6_H_4_	2-F	23	81
13	1	4-MeO-C_6_H_4_	3-F-4-MeO	66	88
14	1	4-MeO-C_6_H_4_	3,4-(CH_2_O_2_)	44	90
15	1	4-MeO-C_6_H_4_	3,4-diMe	36^c^	85
16	1	4-MeO-C_6_H_4_	3,5-diMe	38^c^	90
17	1	4-MeO-C_6_H_4_	3,4,5-triMeO	67	78
18	1	Ph	4-Me	70	87
19	1	4-NMe_2_-C_6_H_4_	4-Me	36	91
20	1	4-F-C_6_H_4_	4-Me	74	89
21	1	4-CF_3_-C_6_H_4_	4-Me	54	90
22	1	3-MeO-C_6_H_4_	4-Me	72	93
23	1	2-MeO-C_6_H_4_	4-Me	28	80
24	1	1*H*-indol-3-yl	4-Me	41	77
25	1	Ph	3-Me	76	88
26	1	Ph	4-MeO	44	80
27	1	4-Me-C_6_H_4_	H	70^c^	88
28	0	4-MeO-C_6_H_4_	4-Me	60	87

^a^5 mol % Pd catalyst were used; ^b^on a 1 mmol scale; ^c^in the presence of 5 equiv H_2_O.

In 2018, the very first heterogeneous catalytic system for the addition of arylboronic acids to cyclic enones was introduced by O’Reilly and co-workers [56]. The micellar nanoreactor was tested for the preparation of flavanones. The main advantages of such catalytic system were short reaction times in an aqueous medium and with a very small amount of the catalyst needed (Table 28). The heterogeneous catalyst **PdL10b** system worked significantly better than the conventional homogeneous synthesis, even when using a significantly higher amount of the **PdL10a** catalytic species in the homogeneous system. The results were excellent both in terms of enantioselectivities and conversions (up to 98%; up to 83% ee; Table 28). The reuse of the heterogeneous catalyst has not been studied in this case.

**Table 28:** Micellar nanoreactor for the synthesis of substituted flavanones [56].

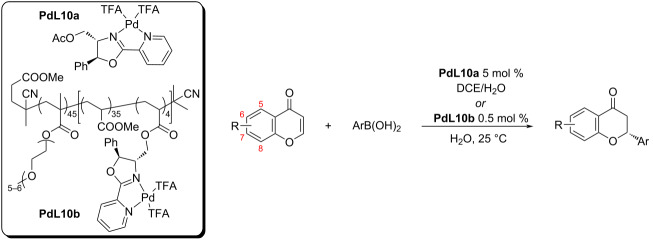									

			homogeneous system with **PdL10a**				heterogeneous system with **PdL10b**		
R	Ar	entry	time (h)	yield (%)	ee (%)	entry	time (h)	yield (%)	ee (%)

H	Ph	1	24	98	84	5	24	90	80
H	Ph	2	24	95^a^	79	6	92	94	82
H	4-Cl-C_6_H_4_	3	24	94	81	7	24	68	76
6-Cl	4-Cl-C_6_H_4_	4	24	80	83	8	24	32	71

^a^30 mol % NH_4_PF_6_.

In 2020, our group reported the first heterogeneous polystyrene-supported recyclable catalyst for the asymmetric conjugate additions of arylboronic acids to five and six-membered enones (Table 29) [57]. For most of the substrates, the enantioselectivity was similar to the values reported for the homogeneous **L9**/Pd(TFA)_2_ system. The conversions obtained were a bit worse, especially for the more sterically demanding boronic acids (Table 29).

**Table 29:** Polystyrene-supported Pd complex **PdL11** as catalyst for addition reactions of arylboronic acids to cyclic enones [57].

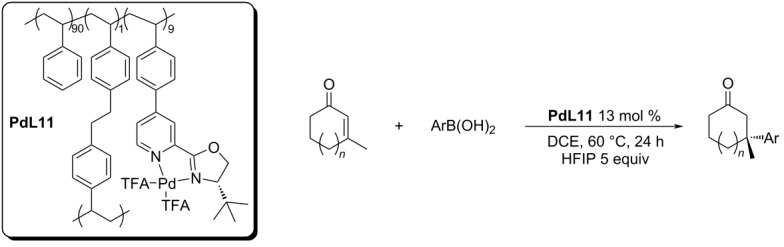				

entry	*n*	Ar	conversion (%)	ee (%)

1	1	Ph	93	89
2	1	4-Me-C_6_H_4_	94	75
3	1	4-CF_3_-C_6_H_4_	85^a^	91
4	1	4-Cl-C_6_H_4_	78^a^	91
5	1	4-Ac-C_6_H_4_	52^a^	90
6	1	4-BnO-C_6_H_4_	59^a^	58
7	0	Ph	99	79
8	0	4-Me-C_6_H_4_	92^a^ (96 h)/99^b^	67/77^b^
9	0	4-MeOOC-C_6_H_4_	99^a^ (96 h)/99^b^	90/89^b^
10	0	3-MeOOC-C_6_H_4_	91^a^ (72 h)/99^b^	91/96^b^

^a^30 mol % NH_4_PF_6; _^b^homogenous conditions: 5 mol % Pd(TFA)_2_, 6 mol % **L9**, 5 equiv H_2_O, 60 °C, 24 h, DCE.

Under the optimised conditions, we were able to use the catalyst in 6 runs with no significant drop in the enantioselectivity and only a small decrease in the conversion (Table 30). The main issues with transferring into heterogeneous conditions were the impossibility of using water as a proton source and the observed reduction of Pd(II) to Pd(0). HFIP was used as a proton source instead and Pd(0) was reoxidised to Pd(II) by *p*-chloranil between the individual cycles. The ratio PS-PyOx:Pd(TFA)_2_ showed a crucial role in the enantioselectivity. Using a higher excess of PS-PyOx allowed achieving a higher ee, however, it also caused a faster loss of catalytic activity.

**Table 30:** Recyclisation of the polystyrene-supported Pd complex **PdL11** [57].

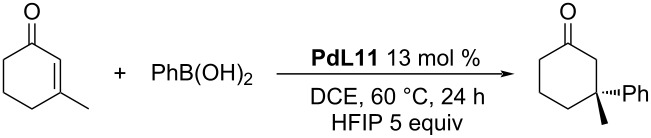							

conversion % (ee %)							
cycle		1st	2nd	3rd	4th	5th	6th

PyOx:Pd ratio	1:2	95 (70)	95 (80)	84 (82)	89 (82)	66 (83)	96 (83)^a^
PyOx:Pd ratio	2:1	93 (89)	54 (90)				
PyOx:Pd ratio	1.3:1	99 (73)	90 (87)^a^	99 (88)^a^	89 (89)^a^	54 (89)^a^	69 (87)^a^

^a^Reoxidation with *p*-chloranil before cycle.

**Scheme 20:**
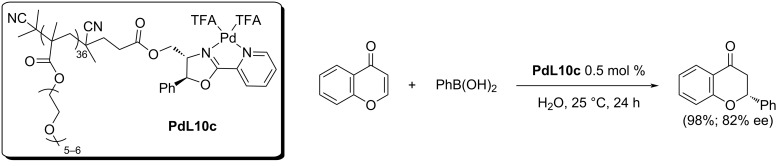
Micellar nanoreactor **PdL10c** for the synthesis of flavanones [58].

Later in 2020, Zhou et al. used an analogous heterogeneous system as O’Reilly (cf. Table 28) [56,58]. A RAFT polymerisation reaction, in this case, led to a polymeric backbone with terminal catalytic centres [58] (Scheme 20). The results obtained were consistent with those reported by O’Reilly using a polymeric backbone with catalytic centres inside the chain [56].

The authors outlined the possibility of recycling the catalyst based on the lower critical solution temperature (LCST) of the catalytic polymer system. The catalyst precipitated and was recovered by centrifugation and discarding the supernatant liquid. This process was complicated by a low catalyst loading and high phase-transition temperature leading to the loss of mass during this procedure. The authors, however, did not try the preparation of a polymer with a lower phase-transition temperature. The loss of mass was compensated by the addition of 10% of fresh catalyst. By this method, they were able to reuse the catalyst in 6 cycles with only a very small decrease in the yield (98, >97, >97, >96, >95, >91%). Unfortunately, the enantioselectivity was not estimated after each cycle [58].

**Scheme 21:**
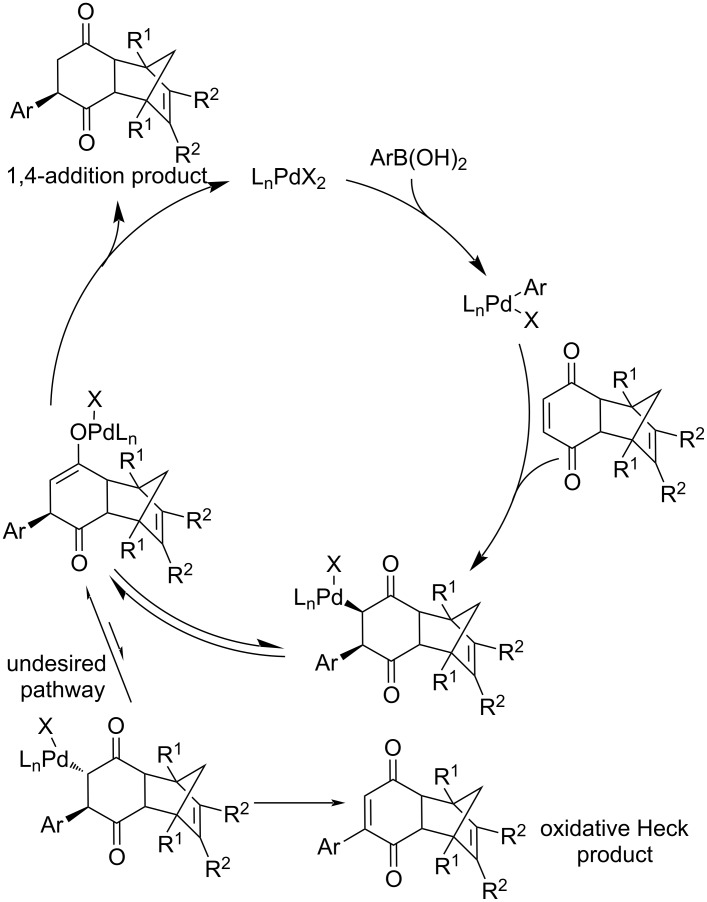
Plausible catalytic cycle for the desymmetrisation of polycyclic cyclohexenediones by the addition of arylboronic acids [59].

In 2019, Lee et al. focused on the enantioselective desymmetrisation of polycyclic cyclohexenediones [59]. The variously substituted pyridine-oxazolines **L9** and **L12a**,**b** were tested as ligands in combination with Pd(OAc)_2_ or Pd(TFA)_2_ (Table 31). As a suitable solvent was chosen DMF, although the use of polar aprotic solvents usually leads to products of the oxidative Heck reaction. The authors noticed a significant reduction of Pd(II) to Pd(0) (by secondary processes such as oxidative homocoupling or oxidation of boronic acid to the corresponding phenol). The Pd(0) reduced in this way was reoxidized to Pd(II) by adding oxygen to the reaction mixture. Excellent enantiomeric excesses were observed (80–96% ee), but the conversions were low (13–83%), especially for boronic acids with electron-acceptor substituents (Table 31). The authors also proposed a plausible catalytic cycle as outlined in Scheme 21 [59].

**Table 31:** Addition reactions of various boronic acids to polycyclic cyclohexenediones [59].

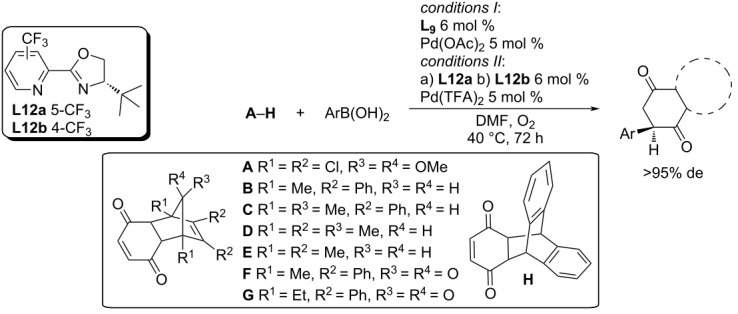					

entry	conditions	substrate	Ar	yield (%)	ee (%)

1	*I*	**A**	4-MeO-C_6_H_4_	80^a^	84
2	*I*	**A**	4-HO-C_6_H_4_	65	80
3	*I*	**B**	4-MeO-C_6_H_4_	70	94
4	*I*	**B**	3-MeO-C_6_H_4_	58	94
5	*I*	**B**	2-MeO-C_6_H_4_	46^b^	84
6	*I*	**B**	4-HO-C_6_H_4_	65	96
7	*I*	**B**	Ph	83^b^	94
8	*I*	**B**	4-Me-C_6_H_4_	81^b^	94
9	*I*	**B**	3-Cl-4-MeO-C_6_H_4_	51^b^	94
10	*I*	**B**	4-F-C_6_H_4_	57^b^ (80)^c^	88
11	*I*	**B**	4-(AcNH)-C_6_H_4_	42^b,d^ (60)^c^	96
12	*I*	**B**	4-EtOOC-C_6_H_4_	13^e^	90
13	*I*	**C**	4-MeO-C_6_H_4_	73^b^	86
14	*IIa*	**D**	4-MeO-C_6_H_4_	64	90
15	*I*	**E**	4-MeO-C_6_H_4_	43^b^	94
16	*IIa*	**E**	4-MeO-C_6_H_4_	68	90
17	*I*	**F**	4-MeO-C_6_H_4_	68	88
18	*I*	**G**	4-MeO-C_6_H_4_	72 (60)^f^	84 (86)^f^
19	*IIb*	**H**	4-HO-C_6_H_4_	65	70

^a^Temperature 30 °C; ^b^**L9** 11 mol % and Pd(OAc_2_) 10 mol %; ^c^NMR yield; ^d^time 92 h; ^e^temperature 50 °C and double amount of catalyst (50% added at the beginning, 50% added after 24 h); ^f^10× larger amount (1 mmol).

The latest ligand derived from pyridine-oxazolines is β-carbolino-oxazoline, whose Pd(II) complex was studied mainly as a catalyst for the addition of arylboronic acids to nitrostyrenes. It also showed to be a highly active catalyst for the addition to enones, under conditions similar to those developed by Stoltz et al. for pyridine-oxazolines (Table 32) [60].

**Table 32:** Addition reactions of arylboronic acids to 3-methyl-2-cyclohexenone catalysed by **L13**/Pd(TFA)_2_ [60].

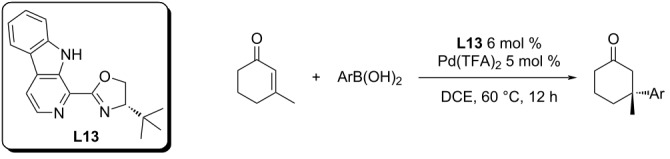			

entry	Ar	yield (%)	ee (%)

1	Ph	88	95
2	4-MeO-C_6_H_4_	75	70
3	4-Me-C_6_H_4_	72	91
4	1-naphthyl	88	89
5	4-CF_3_-C_6_H_4_	86	96
6	4-F-C_6_H_4_	81	95
7	3-Me-C_6_H_4_	73	88
8	3-Cl-C_6_H_4_	88	99

### Catalytic systems based on bisoxazoline ligands

**Scheme 22:**
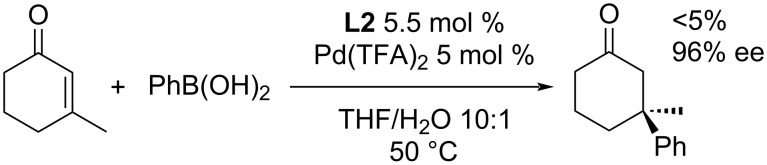
Attempt to use the catalytic system **L2**/Pd(TFA)_2_ for the addition of phenylboronic acid to 3-methyl-2-cyclohexenone [14].

In 2012, the Minnaard group followed up their pioneering work with the phosphine ligand **L2** to expand the substrate scope to 3-substituted enones [14]. At first, they have tried their original catalytic system **L2**/Pd(TFA)_2_ for the addition of phenylboronic acid to 3-methyl-2-cyclohexenone (Scheme 22) that provided the product with an excellent enantioselectivity of 96% but in a very poor yield <5%.

The previously used ligand was changed to bisoxazoline **L14.** At first, they tested in situ-generated complexes of **L14** and Pd(TFA)_2_ in methanol or acetone, but the reduction to catalytically inactive Pd(0) occurred faster. The reoxidation by Cu(BF_4_)_2_·6H_2_O led to the loss of enantioselectivity presumably because of the complexation of the bisoxazoline by Cu(II). This problem could be solved by using a higher amount of the ligand (27 mol %) [14].

The second more favourable solution was the preparation of the bisoxazoline complex with PdCl_2_ followed by dehalogenation. The use of AgSbF_6_ as the dehalogenating agent allowed the complete conversion in the model reaction with a high ee of 96% (entry 3, Table 33). Also the addition reactions to five and six-membered 3-substituted enones proceeded smoothly in most cases (entries 1–11, Table 33), providing the products with remarkable enantioselectivities. The only exceptions were *ortho*-substituted arylboronic acids, which did not react at all (entries 12 and 13, Table 33) [14].

**Table 33:** Addition reactions of arylboronic acids to various enones catalysed by palladium bisoxazoline complex **PdL14** [14].

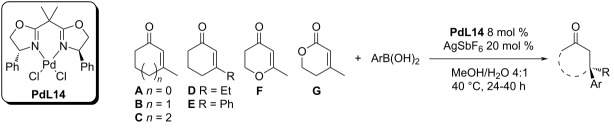				

entry	substrate	Ar	yield (%)	ee (%)

1	**A**	Ph	93	93
2	**A**	4-Me-C_6_H_4_	68	90
3	**B**	Ph	100	96
4	**B**	3-Me-C_6_H_4_	89	97
5	**B**	4-Me-C_6_H_4_	96	97
6	**B**	4-F-C_6_H_4_	88	98
7	**B**	3-EtO-C_6_H_4_	44	93
8	**B**	3-Cl-C_6_H_4_	30^a^	98
9	**B**	3-Cl-4-MeO-C_6_H_3_	98	>99
10	**B**	4-MeO-C_6_H_4_	85	98
11	**B**	3,4-(CH_2_O_2_)-C_6_H_3_	98	96
12	**B**	2-Me-C_6_H_4_	0	–
13	**B**	ferrocenyl	0	–
14	**C**	Ph	80	94
15	**D**	Ph	91	99
16	**E**	Ph	0	–
17	**F**	Ph	28	69
18	**G**	Ph	57	88

^a^60 °C.

A substituent on the enone in position 3 significantly affected the reactivity (entries 3, 15, and 16, Table 33). In the case of dihydropyranone derivatives (entries 17 and 18, Table 33), the reactivity depended on the position of the oxygen in the ring. The tight geminal arrangement of oxygen with the reaction centre reduced the reactivity and enantioselectivity more than in the more distant arrangements. The substrate scope was expanded to 3-substituted linear enones, but the yields were only poor to good (up to 84%) and the enantioselectivities were low to moderate (up to 60% ee; Table 34) [14].

**Table 34:** Addition reactions of arylboronic acids to linear enones catalysed by the bisoxazoline complex **PdL14** [14].

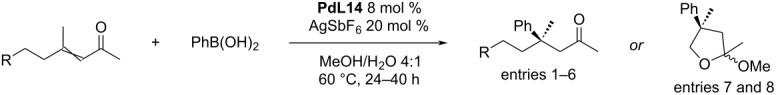				

entry	substrate configuration	R	yield (%)	ee (%)

1	*E*	Ph	14	8
2	*E*	*t*-Bu	<10	–
3	*E*	*t*-BuO	84	23
4	*E*	BnO	81	25
5	*Z*	BnO	78	36
6	*E*	TBDPSO	38	60
7	*E*	TrO	53	51^a^
8	*E*	TIPSO	68	27^a^

^a^Determined after ring opening of the ketal.

**Scheme 23:**
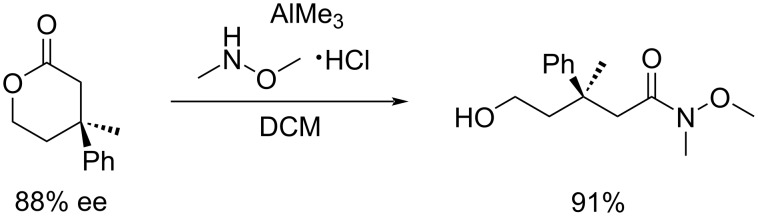
Ring opening of an enantioenriched tetrahydropyran-2-one derivative as alternative strategy to linear products [14].

Another option to obtain the linear product is the ring opening of the addition product of the arylboronic acid to the dihydropyran-2-one derivative (Scheme 23) [14].

The Minnaard group next focused on the increase of the reactivity of *ortho*-substituted boronic acids [14–15]. An optimisation study showed that the presence of AgTFA (dehalogenation reagent) and NH_4_PF_6_ (Pd(II) stabilizing salt) in the reaction mixture was necessary. Additionally, the solvent was changed from a methanol/water mixture to a DCE/water biphasic system. It was also necessary to use a high excess of the starting enone (7 equiv). The results are summarised in Table 35 and it is clear that the yields for most of the cases were very low and exceeded 30% in only a few cases (mostly when a high catalyst amount was used). On the other hand, the enantioselectivities were excellent in almost every example (Table 35) [14–15].

**Table 35:** Addition reactions of *ortho*-substituted arylboronic acids to five and six-membered enones [14–15].

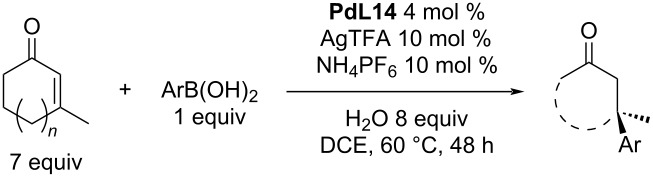				

entry	*n*	Ar	yield (%)	ee (%)

1	0	2-Me-C_6_H_4_	23	90
2	1	2-Me-C_6_H_4_	16	98
3	0	2-MeO-C_6_H_4_	45	80
4	1	2-MeO-C_6_H_4_	42^a^	96
5	0	2-F-C_6_H_4_	20	95
6	1	2-F-C_6_H_4_	23^a^	95
7	0	2-Cl-C_6_H_4_	12^a^	94
8	1	2-Cl-C_6_H_4_	<10	–
9	0	dibenzofuran-4-yl	51	94
10	1	dibenzofuran-4-yl	36	94
11	0	1-naphthyl	38^a^	85
12	1	1-naphthyl	26	95
13	0	2,3-diOMe-5-Me-C_6_H_2_	55^a^	92
14	1	2,3-diOMe-5-Me-C_6_H_2_	19	94
15	0	2,3-diMeO-C_6_H_3_	25	94
16	1	2,3-diMeO-C_6_H_3_	44	99
17	0	2-MeO-5-Me-C_6_H_3_	32^a^	80
18	1	2-MeO-5-Me-C_6_H_3_	28	91
19	0	2,5-diMeO-4-Me-C_6_H_2_	21^a^	74
20	1	2,5-diMeO-4-Me-C_6_H_2_	<10	84
21	0	2-MeO-4-Me-C_6_H_3_	<10	68
22	1	2-MeO-4-Me-C_6_H_3_	17	90

*8 mol % **PdL14** used.

**Scheme 24:**
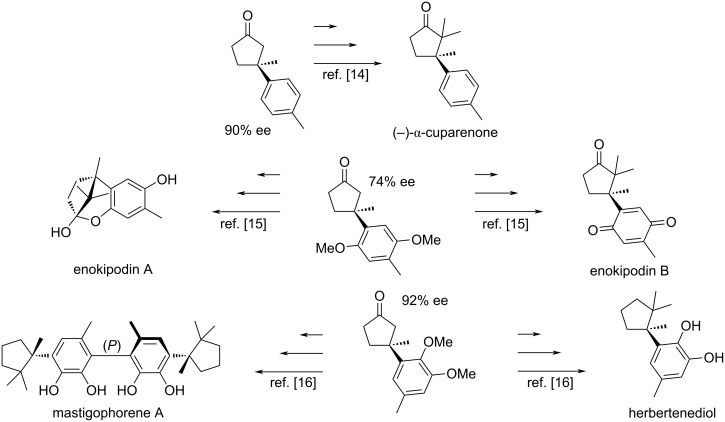
Synthesis of biologically active compounds from addition products [14–16].

Selected addition products were used as intermediates in the total syntheses of various biologically active compounds (Scheme 24) [14–16].

### Catalytic systems based on different groups of ligands

**Scheme 25:**
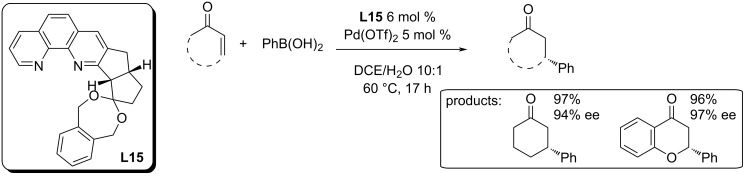
Chiral 1,10-phenantroline derivative **L15** as ligand for the Pd-catalysed addition reactions of phenylboronic acid to 2-cyclohexenone and chromone [61].

The use of the chiral 1,10-phenanthroline ligand **L15** for the addition of phenylboronic acid to 2-cyclohexenone and chromone (Scheme 25) [61] was proposed by Tamura et al. in 2017. Excellent conversions and enantioselectivities (96–97%; 94–97% ee) were achieved for both studied substrates. However, a further use of this ligand has not been published yet.

Optically pure pyridine-hydrazones were successfully used for a number of various enantioselective transformations [62]. In 2019, Retamosa et al. used them for 1,4- and 1,6-addition reactions of boronic acids to cyclic (di)enones. Initial studies showed the best yields when DCE was used as a solvent upon the addition of 0.2 equiv of water [62]. Without the addition of water, no reproducible results were obtained. The addition of 1.1–1.5 equiv of water caused a minimal decrease of the enantioselectivity from 91 to 88% ee (entries 1 and 2, Table 36) [62].

**Table 36:** Addition reactions of arylboronic acids to five and six-membered enones catalysed by **L16**/Pd(TFA)_2_ [62].

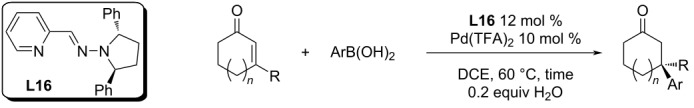						

entry	*n*	R	Ar	time (h)	yield (%)	ee (%)

1	1	Me	Ph	24	94	91
2	1	Me	Ph	24	90^a^	88^a^
3	1	Me	4-Me-C_6_H_4_	48	93	91
4	1	Me	4-F-C_6_H_4_	72	43	90
5	1	Me	4-Cl-C_6_H_4_	72	77	90
6	1	Me	4-MeO-C_6_H_4_	72	73	90
7	1	Me	4-CF_3_O-C_6_H_4_	72	65	90
8	1	Me	3,5-diMe-C_6_H_3_	24	75	92
9	1	Et	Ph	48	80	89
10	1	Ph	4-MeO-C_6_H_4_	72	0	–
11	1	H	Ph	48	76	87
12	0	Me	Ph	20	95	88
13	0	Me	2-MeO-C_6_H_4_	48	73	91
14	0	Me	4-Me-C_6_H_4_	48	97	88
15	0	Me	3,4-(CH_2_O_2_)C_6_H_3_	60	65^b^	86^b^
16	0	Me	2,5-diOMe-4-MeC_6_H_2_	72	38	93

^a^1.1 equiv of water used; ^b^**L16** 9 mol % and Pd(TFA)_2_ 7.5 mol % were used.

For the whole series of different substrates and boronic acids, there were enantioselectivities of about 90% ee and average to excellent yields of 43–97% (Table 36) [62]. This catalytic system worked for 3-unsubstituted enones but was much more powerful in the case of addition reactions to 3-substituted enones that lead to all-carbon quaternary stereogenic centres [62].

In the case of 1,6-additions, the amount of the starting dienones was increased to 4.17 equivalents relative to the boronic acids. Further, the boronic acid was gradually added over 12 hours and then the mixture was kept under the reaction conditions for another time period up to total 72 or 96 h. The prolonged reaction time increased the obtained yields but at the expense of reducing the enantioselectivity of the product (61 to 81%; 79 to 67% ee; entries 1 and 2, Table 37). This led to the conclusion that the ligand is not chemically stable in the reaction medium and undergoes decomposition over time. Only low to average conversions (up to 81%) and only average enantioselectivities (up to 80% ee; Table 37) were achieved for the studied substrates [62].

**Table 37:** 1,6-Addition reaction of arylboronic acids to dienones catalysed by **L16**/Pd(TFA)_2_ [62].

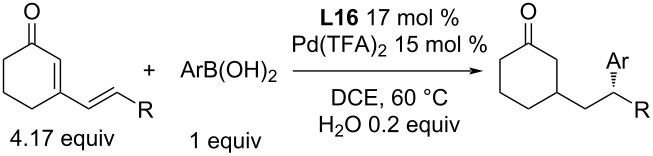					

entry	R	Ar	time (h)	yield (%)	ee (%)

1	Me	Ph	72	61	79
2	Me	Ph	96	81	67
3	Me	4-Me-C_6_H_4_	72	44	74
4	Me	4-Me-C_6_H_4_	96	78	68
5	Me	4-CF_3_O-C_6_H_4_	72	35	80
6	Me	4-CF_3_O-C_6_H_4_	96	47	72
7	*n-*Bu	Ph	72	31	52

One of the most recent contributions to this topic came from the group of Hong and Stoltz in 2020. Here, attention was focused on the development of a methodology for the enantioselective addition to 2-substituted chromones [63]. The original work from the Stoltz group using pyridine-oxazolines was very successful for addition reactions to 2-unsubstituted chromones (Table 23). However, in the attempted addition reaction of phenylboronic acid to 2-methylchromone, the expected product was not isolated (entry 6, Table 22) [51]. Therefore, a new optically pure substituted pyridine-dihydroisoquinoline **L17** was developed (Table 38) [63]. The studied catalytic system of ligand **L17** in combination with Pd(TFA)_2_ allowed the isolation of the desired products in excellent yields, especially for electron-rich boronic acids. The yields for the products from addition reactions with electron-poor boronic acids were only average. However, excellent enantioselectivities were achieved for all studied substrate combinations (90–99% ee; Table 38) [63].

**Table 38:** Addition reactions of arylboronic acids to 2-substituted chromones catalysed by **L17**/Pd(TFA)_2_ [63].

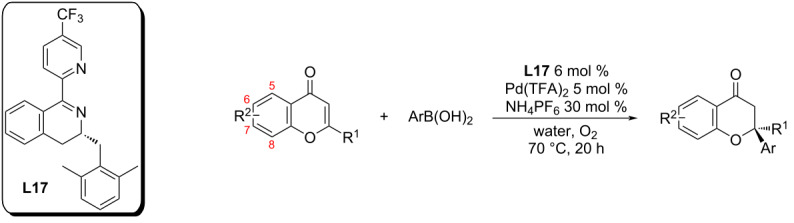					

entry	R^1^	R^2^	Ar	yield (%)	ee (%)
1	Me	H	Ph	98	95
2	Me	H	4-Me-C_6_H_4_	80	96
3	Me	H	4-Et-C_6_H_4_	85	98
4	Me	H	4-MeO-C_6_H_4_	51	90
5	Me	H	4-*t-*Bu-C_6_H_4_	78	98
6	Me	H	3-MeO-C_6_H_4_	81	99
7	Me	H	3-Me-C_6_H_4_	82	99
8	Me	H	3,5-diMe-C_6_H_3_	77	97
9	Me	H	3,4-(CH_2_O_2_)-C_6_H_3_	47	96
10	Me	H	4-F-C_6_H_4_	80	98
11	Me	H	4-Cl-C_6_H_4_	86	99
12	Me	H	4-Br-C_6_H_4_	32	98
13	Me	H	4-CF_3_-C_6_H_4_	31	99
14	Me	H	3-F-C_6_H_4_	60	96
15	Me	H	3-Cl-C_6_H_4_	55	92
16	Et	H	Ph	93	98
17	iPr	H	Ph	47	97
18	Cy	H	Ph	48	98
19	Bn	H	Ph	52	98
20	Me	6-Me	Ph	89	98
21	Me	6-MeO	Ph	88	98
22	Me	7-MeO	Ph	92	98
23	Me	6-F	Ph	74	97
24	Me	6-Cl	Ph	90	96
25	Me	6-Br	Ph	64	99

### Evaluation of current state and outlook

**Scheme 26:**
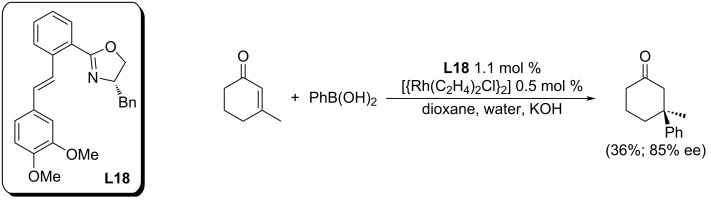
The Rh-catalysed addition reaction of phenylboronic acid to a 3-substituted enone [20].

Asymmetric addition reactions to enones have so far been described in the literature in connection with catalysis. The catalyst is usually a complex of a transition metal with a suitable ligand. However, metal-free catalysis is also known [64]. Among the most successful transition-metal catalysts are those based on rhodium, as evidenced by the number of reports that deal with the issue. The rhodium-catalysed addition of various boronic acids to conjugated cyclic enones (the so-called Hayashi–Miyaura reaction) is a well-established method for 3-unsubstituted substrates as well as for 2-unsubstituted chromones [17–19,21–24]. On the other hand, there is only one example of the usage of a rhodium-based catalyst for the addition of arylboronic acid to 3-substituted enones. The olefino-oxazoline ligand **L18** has been used for the rhodium-catalysed addition reaction of phenylboronic acid to 3-methyl-2-cyclohexenone and affording the product in a low yield and moderate enantioselectivity (36%; 85% ee; Scheme 26) [20]. Palladium-based catalysis provides better results in this area.

**Scheme 27:**
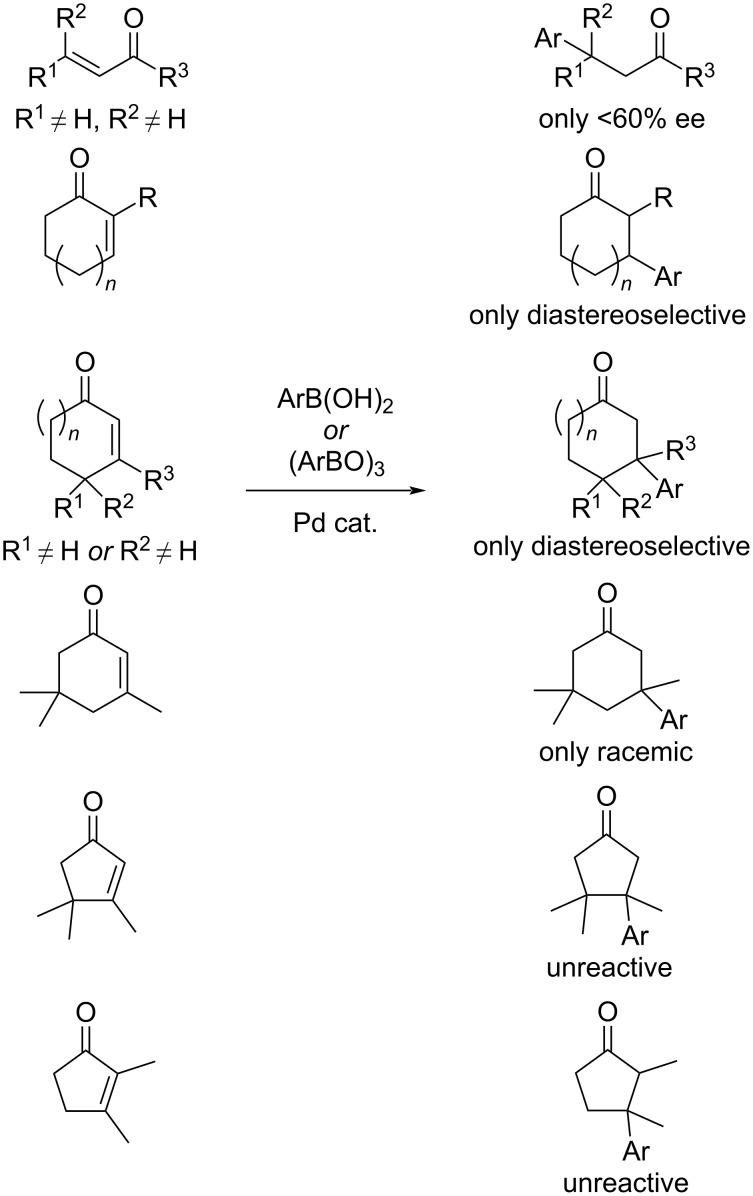
Underdeveloped methodologies [14–15,65–67].

Up to now, asymmetric addition reactions to sterically hindered enones are still challenging. In Scheme 27, we present some underdeveloped methodologies.

We have so far tried to achieve asymmetric addition to some of these cyclic enones in our laboratory without success. Specifically, it was catalysis in a homogeneous medium, using ligand **L9** and Pd (TFA)_2_. Also, continuous-flow reactions are currently a general challenge, especially for the pharmaceutical industry. The prerequisite for a successful continuous synthesis in the field of asymmetric addition reactions to enones is the mastery of recyclable heterogeneous catalysis. Very recently, we reported [57] the first heterogeneous polystyrene-supported recyclable catalyst for asymmetric conjugate addition reactions of arylboronic acids to five and six-membered enones. In our laboratory, we also attempted to perform this reaction under flow conditions. However, the change from batch to flow arrangement itself is another challenging task. Nevertheless, it should be noted at this point that in the case of rhodium complex catalysis, the asymmetric addition of phenylboronic acid to enones in continuous flow has been successful [24]. In 2021, Walhers et al. presented a theoretical study based on the Q2MM method about the asymmetric addition of arylboronic acids to conjugated cyclic enones, catalysed by a complex of **L9** and Pd(TFA)_2_ [68]. The authors prepared a training set from the data of currently known combinations of PyOx derivatives as ligands, boronic acids and enones (82 hits). They have calculated the predictions of enantioselectivities for Pd(TFA)_2_ complexes of 27 new PyOx-type ligands (for the reaction of 3-methyl-2-cyclohexenone with phenylboronic acid) and 59 new enones (in reactions with phenylboronic acid catalysed by **L9**/Pd(TFA)_2_). The calculation performed was related to a transition state and included steric and inductive effects. Although this approach may be suitable for predicting theoretically achievable enantioselectivity and is very promising, it is not engineered to predict reactivity. Besides, the reactivity (conversion or yield) depends on the reaction medium which is not included in the theoretical model. The experimental validation of the predicted results is therefore a challenge that has to be finished [68].

## Conclusion

**Scheme 28:**
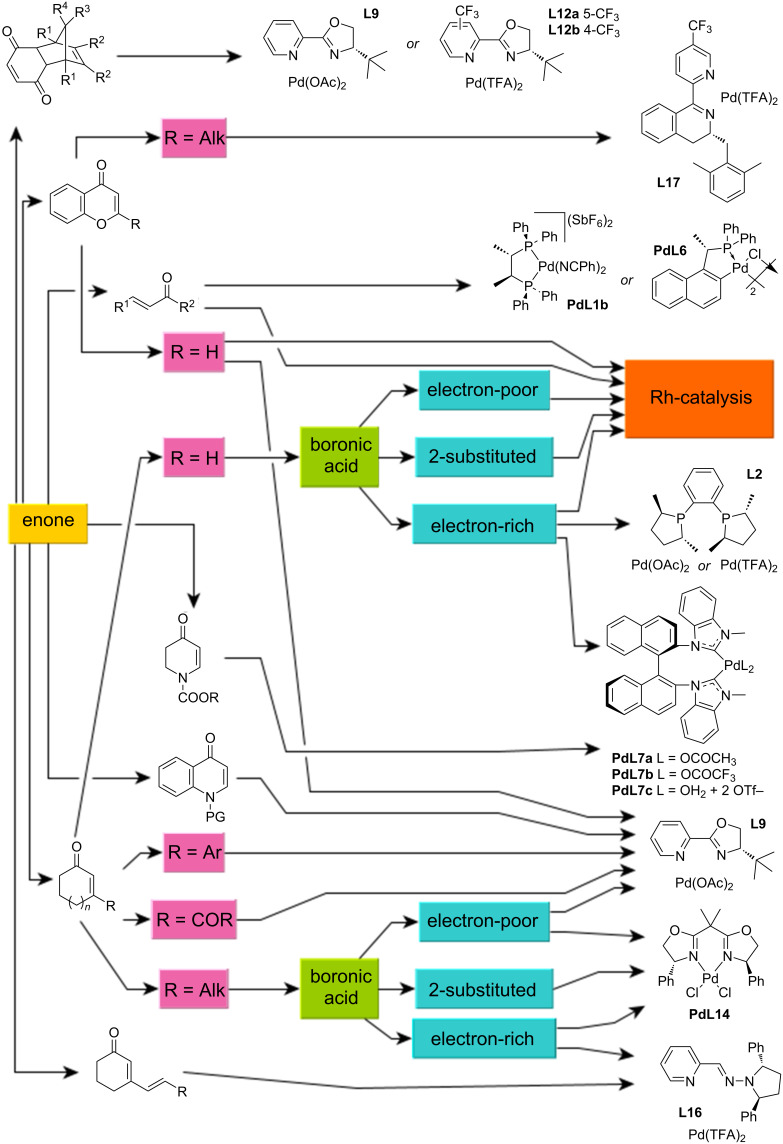
Flowchart for the selection of the proper catalytic system.

In this review, we focused on palladium-catalysed asymmetric 1,4-addition reactions of arylboronic acids to conjugated enones and chromones. The suitability of the ligand used, the reaction conditions, and additives in terms of the yield and enantioselectivity of the transformation have been discussed. The review is classified according to the type of ligand of the catalytic complex used. The yields and corresponding enantioselectivities from the relevant literature were summarised in clear tables. Based on the above results, we propose a flowchart facilitating the reader in selecting a suitable ligand for a given combination of enone and arylboronic acid (Scheme 28). However, the reader should be aware of its limitations because not all ligands have been studied on all substrates. Also, close to the end of the review, the catalysis by rhodium complexes has been mentioned. With these catalysts only reactions of 3-unsubstituted enone derivatives have been described. It can be said that, despite great efforts, some problems remain unresolved. Thus, palladium-based catalysts represent a more suitable alternative to the widely used rhodium complexes for these sterically hindered enone derivatives.
